# Variance-Preserving Estimation of Intensity Values Obtained From Omics Experiments

**DOI:** 10.3389/fgene.2019.00855

**Published:** 2019-09-20

**Authors:** Adèle H. Ribeiro, Julia Maria Pavan Soler, Roberto Hirata

**Affiliations:** ^1^Department of Computer Science, Institute of Mathematics and Statistics, University of São Paulo, São Paulo, Brazil; ^2^Department of Statistics, Institute of Mathematics and Statistics, University of São Paulo, São Paulo, Brazil

**Keywords:** delta method, pixel-level uncertainty, spot quantification, optimal LOWESS normalization, two-color microarray, variability preservation, parameter selection

## Abstract

Faced with the lack of reliability and reproducibility in omics studies, more careful and robust methods are needed to overcome the existing challenges in the multi-omics analysis. In conventional omics data analysis, signal intensity values (denoted by *M* and values) are estimated neglecting pixel-level uncertainties, which may reflect noise and systematic artifacts. For example, intensity values from two-color microarray data are estimated by taking the mean or median of the pixel intensities within the spot and then subjected to a within-slide normalization by LOWESS. Thus, focusing on estimation and normalization of gene expression profiles, we propose a spot quantification method that takes into account pixel-level variability. Also, to preserve relevant variation that may be removed in LOWESS normalization with poorly chosen parameters, we propose a parameter selection method that is parsimonious and considers intrinsic characteristics of microarray data, such as heteroskedasticity. The usefulness of the proposed methods is illustrated by an application to real intestinal metaplasia data. Compared with the conventional approaches, the analysis is more robust and conservative, identifying fewer but more reliable differentially expressed genes. Also, the variability preservation allowed the identification of new differentially expressed genes. Using the proposed approach, we have identified differentially expressed genes involved in pathways in cancer and confirmed some molecular markers already reported in the literature.

## Introduction

The growing number of omics datasets (e.g., genomics, transcriptomics, proteomics, metabolomics) and the recent advances in multi-omics integration approaches have contributed to the better understanding of biological mechanisms and also the emergence of the personalized medicine. However, the lack of reliability and reproducibility in omics studies stands as one of the biggest obstacles in bridging the gap between research and practice of personalized medicine (Alyass et al., 2015; Karczewski and Snyder, 2018). Considering that inflated variability and non-robust estimation may lead to inaccurate and misleading results, this paper proposes improvements to the conventional estimation and normalization of the intensity values obtained from omics experiments. Specifically, the proposal is to estimate the intensity values by a method that accounts for the variability due to pixel-level uncertainties and to normalize these values by using LOWESS with suitably selected parameter values, preserving variation that may be relevant to subsequent analyses.

Image processing and fluorescence analysis are the preferred approaches for data quantification in microarray technologies. Although microarrays have been predominantly used since the end of the nineties to measure gene expression levels, they remain widely used to detect other omics data types, including microRNA expression, DNA methylation, single-nucleotide polymorphisms (SNPs), and copy number variants (CNVs) (Goodwin et al., 2016). After hybridization and cleaning of the target molecules, the array is scanned by activation with lasers at different wavelengths (one for each of the fluorophores used), and each laser channel generates an image. The pixel intensities within each spot in these microarray images are summarized to represent the hybridization signal. Depending on the platform (e.g., gene expression array, DNA methylation array, SNP array, and comparative genomic hybridization [CGH] array), the interpretation of this signal is different (e.g., gene expression levels, methylation levels, allele frequencies, and copy number alterations).

The continuance of the microarray technology can be mainly explained by the availability of many datasets in public repositories, such as the Gene Expression Omnibus (GEO) (Edgar et al., 2002; Barrett et al., 2012) and ArrayExpress (Kolesnikov et al., 2015), by the existence of well-established strategies for data analysis and experimental design, and by the low cost compared with the next-generation sequencing technologies. However, given that microarray analysis is still facing reliability and reproducibility problems, more robust and rigorous methods are needed to account for the high variability and biases introduced in all steps of a microarray experiment.

Several preprocessing and normalization procedures have been proposed to remove biases due to the inhomogeneity of the background and the different fluorescence properties of the dyes. However, biases introduced in the image analysis step, which includes spot segmentation and signal extraction, have not received the same attention, and those may partially explain the existing reliability and reproducibility problems in omics studies. Particularly, several factors, including image resolution, scanner settings, effectiveness of the segmentation algorithm, and unexpected behaviors during hybridization, may lead to errors in spot localization and classification of the pixels (as foreground or background, depending on whether it is situated within or around the spot). Thus, spot intensities are usually noisy and that high pixel–level variability leads to uncertainty in microarray quantification and correlates with variability between replicate spots on duplicate slides (Brown et al., 2001).

Given that even state-of-art image processing tools are susceptible to errors that significantly influence the variability of the data derived from microarray images (Ahmed et al., 2004), new segmentation and intensity extraction algorithms are still being developed in order to improve precision in spot quantification (Li et al., 2017; Karthik and Manjunath, 2018; Shao et al., 2019). Usually, these tools combine sophisticated algorithms and pixel-level analyses in order to obtain an accurate estimate of the signal intensity in each spot. However, to allow subsequent analyses to take into account possible errors and uncertainties arising from the image processing, the method output usually includes not only statistical measures of location (e.g., mean and median) of the foreground and background intensities of each channel of each spot but also measures of dispersion, including standard deviation and covariance between both channels.

Despite the common use of pixel-level variability measures as data quality criteria for filtering purpose, the conventional microarray analysis is solely based on statistical measures of location of the spot intensities (Yang et al., 2002; Sun et al., 2011; Brady and Vermeesch, 2012). To improve robustness and reliability in microarray analysis, pixel-level uncertainties should be accounted for in the intensity log-ratio estimation and propagated to the next steps of the analysis.

Pixel-level uncertainties have been taken into account by many spot quantification algorithms in the literature, but requiring all pixel values to be available. Some of them are interested in improving the log-ratio estimator. Particularly, the method proposed by (Dodd et al., 2004) is a log-ratio estimator that corrects for signal saturation by regressing all pixel intensities at both test and control channels using a censored regression model. The META algorithm (Chan and Chang, 2009) estimates the intensity log-ratio by grouping the pixels according to their distance to the center of the spot and then weighting the log-ratio of each group in inverse proportion to its sample variance. A method that only uses pixel-level mean and variance summary statistics is the hierarchical maximum-likelihood estimator (Bakewell and Wit, 2005). However, it is not exactly based on the standard log-ratio representation of the spot intensity. It models the gene expression signal at control and treatment channels separately, incorporating the sample within-spot deviation and then performs the estimation using maximum likelihood. To the best of our knowledge, there is no intensity log-ratio estimator to be used after the image analysis phase (i.e., based solely on the pixel-level summary statistics) that takes into account pixel-level uncertainties.

The first contribution of this paper is a more robust estimator for the intensity log-ratio (*M*) and average log intensity (*A*) of a microarray spot that accounts for pixel-level variance and covariance between channels. For a spot *t*, these values are denoted by *M_t_* and *A_t_*, respectively (Dudoit et al., 2002). We derive these estimators by using the multivariate delta method (Casella and Berger, 1990). Specifically, we approximate the expected values of *M_t_* and *A_t_* by using their second-order Taylor’s expansions, and the variance of *M_t_* and *A_t_* by using their first-order Taylor’s expansions. These expansions depend on the pixel-level variance and covariance between channels of the spot, whose sample estimates are readily accessible through standard output files of microarray image analysis tools.

After spot intensity estimation, it is necessary to perform a within-slide normalization to remove array-specific effects, intensity-dependent dye biases, and other systematic trends of the microarray data. The within-slide normalization based on the robust locally weighted regression (LOWESS) (Cleveland, 1979) is one of the most used techniques. The choice of the LOWESS parameters, particularly the smoothing parameter (also known as neighborhood size or bandwidth), dramatically affects the intensity and quality of the microarray data calibration. Although the smoothing parameter is still commonly set arbitrarily (around 0.2 and 0.4) (Dudoit et al., 2002; Smyth and Speed, 2003; Drăghici, 2012), some data-driven methods have been proposed to select its optimal value (Berger et al., 2004; Futschik and Crompton, 2004a; Lee et al., 2008). All these methods are similar in that they choose the smoothing parameter by minimizing a measure of error of the LOWESS fit. Berger et al. (2004) use the mean-squared difference between the LOWESS estimates and the corresponding normalization reference levels as cost function. These normalization levels are the true spot-specific calibration errors, which are usually unknown. Thus, Berger et al. suggest to estimate them from control transcripts and replicate slides. However, they are not always available for all genes in a typical microarray experiment, making it hard to reliably use the method. Futschik and Crompton’s selection method, named OLIN (Futschik and Crompton, 2004a; Futschik and Crompton, 2004b), has the advantage of not relying on a reference level. Its optimization procedures use the generalized cross-validation (GCV) criterion, an estimator of the prediction mean square error (PMSE), as cost function. Lee et al. (2008) proposes to select the smoothing parameter by minimizing the bootstrap estimate of the mean integrated square error (MISE) and show that their results are comparable to OLIN.

Although all these methods have shown superiority over LOWESS normalization with a fixed arbitrarily chosen smoothing parameter, they lack in taking into account any heteroskedasticity in the data. In addition, they usually suffer from a poor bias–variance trade-off, tending to choose small smoothing values, which yield unnecessarily complicated (with high variance) LOWESS fits.

The second contribution of this paper is a data-driven method for selecting the smoothing parameter of the LOWESS normalization process. Inspired by the previous proposed methods, we choose the optimal smoothing value by minimizing a mean squared error criterion. However, our selection method also takes into account heteroskedasticity of the microarray data and offers a better bias–variance trade-off by selecting from among the low-MSE fits the one that is the most parsimonious. The parameter selection is obtained by solving a discrete optimization problem and is based on conventionally accepted ideas for analysis of M-plots—a graphical tool showing the curve of the MSE against the effective degrees of freedom of the estimate (Cleveland et al., 1988).

Given that the primary application of DNA microarrays has been to measure gene expression levels, we focus in this paper on variation-preserving estimation and normalization methods for gene expression levels from two-channel (or two-color) microarrays. However, it is straightforward to adapt the same ideas to improve analysis of other types of microarray data, even from single-channel technologies.

The proposed methods were evaluated by a differential gene expression analysis from real intestinal metaplasia and normal microarray samples. The proposed estimators for the *M_t_* and *A_t_* values were compared with the conventional estimators that neglect the pixel-level variability. In addition, we compared the proposed method for selecting the LOWESS smoothing parameter with OLIN, as it is conceptually similar to the other existing methods and can be applied even to microarray experiments with few or no replicates. Results show that a more robust and conservative analysis is performed when the LOWESS smoothing parameter is selected by our method, potentially reducing the number of false-positive differential expressions. Besides, both the pixel-level variabilities incorporated by the proposed estimators for the *M_t_* and *A_t_* values and the variability preserved by our more parsimonious normalization method contributed to the identification of new differentially expressed genes. Thus, the proposed methods may also reduce the false-negative rate.

## Materials and Methods

Two procedures that critically affect the adequacy of microarray data analysis are the spot quantification, which extracts summarized quantitative measures of the pixel intensities within each spot of the microarray slide, and the within-slide normalization, which removes dye-specific biases and other systematic noises simultaneously from all logged spot intensities (*M_t_* and *A_t_* values).

In the section Intestinal Metaplasia Database, we describe a gene expression dataset used to illustrate the application of our proposed methods. In the section Improved Estimators for the *M_t_* and *A_t_* values, we show our improved estimation method for the *M_t_* and *A_t_* values that incorporates pixel-level variability. In the section Estimators for the Variances of the *M_t_* and *A_t_* Values, we discuss some criteria that can be used for proper setting of the parameters of the LOWESS within-slide normalization and we propose an algorithm for selecting the optimal value for the smoothing parameter (denoted by *f*).

### Intestinal Metaplasia Database

Due to a chronic inflammatory process, the normal squamous mucosa of the stomach may be replaced by columnar intestinal-type epithelium, characterizing a disease called intestinal metaplasia of the stomach. Since adenocarcinoma of the stomach and inflamed intestinal mucosa are strongly associated (Coussens and Werb, 2002), intestinal metaplasia may be a significant risk factor for gastric cancer.

We analyzed data from a two-color microarray experiment with tissues samples from 90 different subjects, being 35 from tissues representing type II intestinal metaplasia and 55 from tissues representing the normal condition, obtained from the Tumor Bank at A.C. Camargo Cancer Center/Antonio Prudente Foundation.

It was used the standard reference design (Churchill, 2002), in which each sample is hybridized against a pool of normal tissues using the same orientation of dye labeling. Gene expression levels were measured on Agilent Whole Human Genome Microarrays 4x44K G4112F (design ID 014850), each slide containing 41,093 unique probes. The scanned images of the microarray slides were processed by *Agilent Feature Extraction* software, version 9.5, where statistics (mean, standard deviation, and covariance) of the foreground and local background pixels were computed for each spot, in both test and reference channels. Each microarray spot contains about 60 foreground pixels.

This study was carried out in accordance with the recommendations of the international guidelines for investigations involving human beings with written informed consent from all subjects. All subjects gave written informed consent in accordance with the Declaration of Helsinki. The protocol was approved by the Ethics Institutional Committee of the A.C. Camargo Cancer Center (process number 1023/07).

### Improved Estimators for the *M_t_* and *A_t_* Values

Usually, in microarray analysis, the test channel is denoted by (red), and the reference channel is denoted by *G* (green), following this usual notation, denoted by *R_tj_* and by *G_tj_*, the intensity value of the *j*th pixel within the th spot, respectively, in the test and reference channel. The relative expression of pixel *j* within spot is denoted by *M_tj_* and defined as follows:

(1) $$M_{tj}≐\log_{2}\left(\frac{R_{tj}}{G_{tj}}\right)=\log_{2}(R_{tj})-\log_{2}(G_{tj}).$$

The average expression of pixel within spot is denoted by *A_tj_* and defined as follows:

(2) $$A_{tj}≐\frac{1}{2}\left(R_{tj}G_{tj}\right)=\frac{\log_{2}(R_{tj})+\log_{2}(G_{tj})}{2}.$$

Usually, image analysis software does not provide all pixel intensity values within each spot. Nonetheless, it provides several descriptive statistics of the foreground and background pixel intensities, including sample estimates for the mean, median, variance, and covariance between the two channels.

To incorporate the pixel-level variability in the analysis, we derived an approximation of the expected values of *M_tj_* and *A_tj_* by using the *multivariate delta method* (Casella and Berger, 1990). Assuming that the functions (1) and (2) are twice differentiable on an open interval which contains the point $\left(E(R_{tj}),E(G_{tj})\right)$, we computed their second-order Taylor’s expansions, around the point $\left(E(R_{tj}),E(G_{tj})\right)$, and then derived their expected values. The derivation is presented in Appendix 4.

It is reasonable to assume that the variables *R_tj_*, *G_tj_*, *M_tj_* and *A_tj_* have a distribution with well-defined mean and variance. Particularly, Hoyle et al. (Hoyle et al., 2002) empirically showed that the distribution of the pixels within a spot is heavy-tailed (a non-Gaussian distribution) and well-approximated by a log-normal distribution. Consequently, *M_tj_* and *A_tj_* follow a distribution which is well-approximated by a Gaussian distribution and all the variables have at least the first and second moments finite.

Let ${\bar{R}}_{tc}$ and ${\bar{G}}_{tc}$ be non-zero estimates of, respectively, $E(R_{tj})$ and $E\;(G_{tj})$, which represent average foreground signals after correction for removing the background influence. The subscript indicates dependence on the background correction. Also, let ${\hat{\sigma }}^{2}(R_{t})$ and ${\hat{\sigma }}^{2}(G_{t})$ be estimates of, respectively, Var (*R_tj_*) and Var (*G_tj_*), which are assumed to be independent of the background correction. Note that mean and variance estimates are calculated across observed foreground pixel intensities within the spot at the respective channel.

We can derive improved estimators for $E\;(M_{tj})$ and $E\;(A_{tj})$ as follows:

(3) $$\begin{array}{l} {\tilde{M}}_{t}≐E(M_{tj})\approx \log_{2}({\bar{R}}_{tc})-\log_{2}({\bar{G}}_{tc})\\ \;+\frac{1}{2ln\;(2)}\left(-\frac{{\hat{\sigma }}^{2}(R_{t})}{{\bar{R}}_{tc}^{2}}+\frac{{\hat{\sigma }}^{2}(G_{t})}{{\bar{G}}_{tc}^{2}}\right), \end{array}$$

(4) $$\begin{array}{l} {\tilde{A}}_{t}≐E(A_{tj})\approx \frac{1}{2}\left(\log_{2}({\bar{R}}_{tc})+\log_{2}({\bar{G}}_{tc})\right)\\ \;-\frac{1}{4ln\;(2)}\left(\frac{{\hat{\sigma }}^{2}(R_{t})}{{\bar{R}}_{tc}^{2}}+\frac{{\hat{\sigma }}^{2}(G_{t})}{{\bar{G}}_{tc}^{2}}\right). \end{array}$$

Note that the conventional estimators for the *M_tj_* and *A_tj_* values, given by

(5) $${\hat{M}}_{t}≐\log_{2}({\bar{R}}_{tc})-\log_{2}({\bar{G}}_{tc}),$$

(6) $${\hat{A}}_{t}≐\frac{\log_{2}({\bar{R}}_{tc})+\log_{2}({\bar{G}}_{tc})}{2},$$

are approximations of, respectively, $E\;(M_{tj})$ and $E\;(A_{tj})$ derived from only the zeroth-order Taylor’s expansion of the functions that define *M_tj_* and *A_tj_*. Thus, the conventional estimators ignore the known measures of pixel-variability, which represent uncertainties in the gene expression measurements.

**Figure 1:**
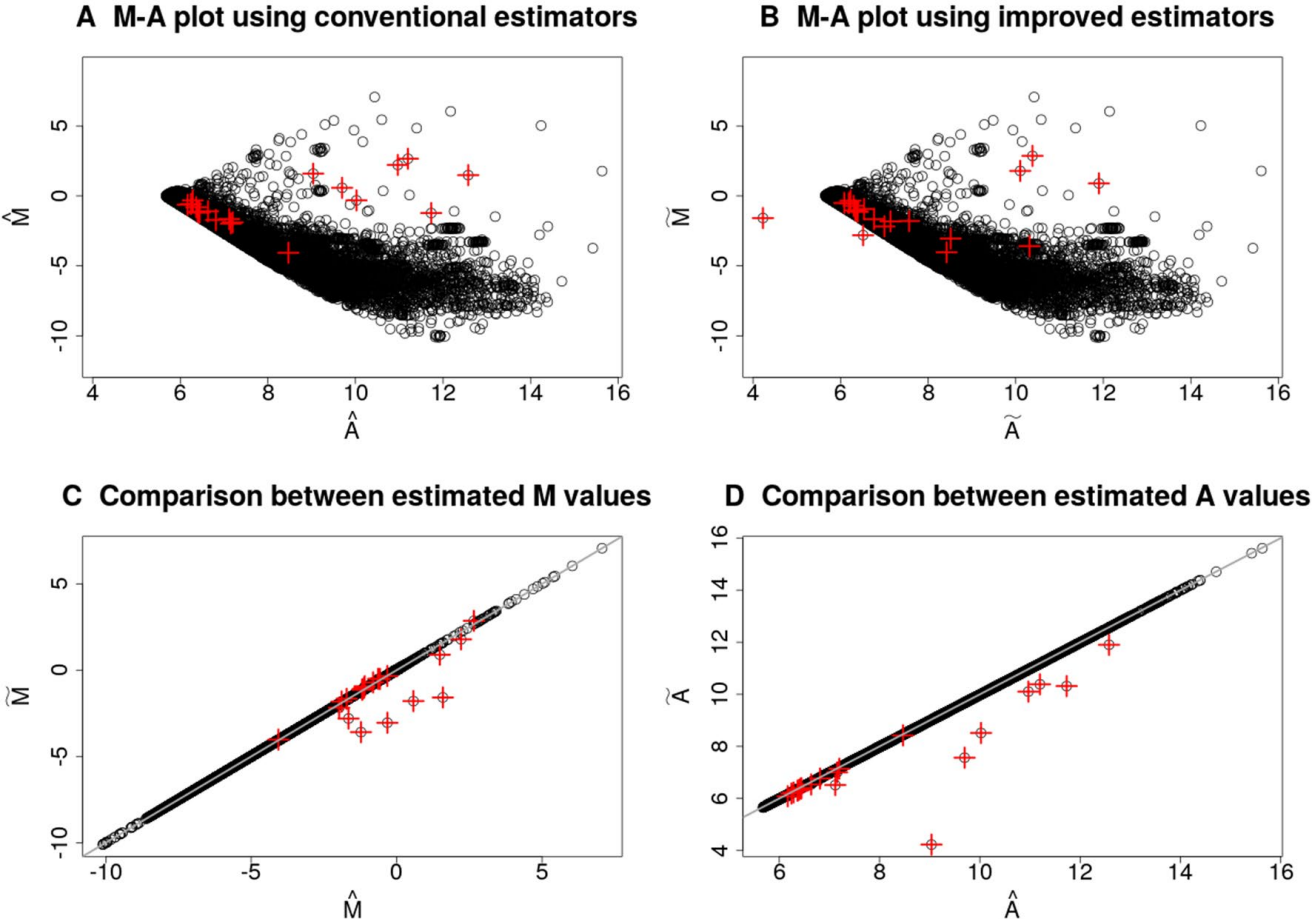
Comparison between conventional and proposed estimation methods for the *M_t_* and *A_t_* values for the microarray slide with ID 251485069395_1.4. The M-A plots in **(A)** and **(B)** were obtained by using, respectively, the conventional and improved estimators for the *M_t_* and *A_t_* values. Plots **(C)** and **(D)** show the conventional against the improved estimates for, respectively, the *M_t_* and *A_t_* values. Top 20 genes with the highest pixel-level variance are highlighted in red plus symbols. The test channel contains RNA samples of normal gastric mucosa, and the control channel contains samples from a common reference. The background influence was removed from the foreground signals by the *normexp* method with offset.

Figure 1 illustrates the differences between the estimators for the $E\;(M_{tj})$ and $E\;(A_{tj})$ for a randomly chosen microarray slide of the database described in the section *Intestinal Metaplasia Database*. Since these estimators may suffer from numerical instability if the corrected foreground signals, ${\bar{R}}_{tc}$ and ${\bar{G}}_{tc}$, are very close to zero, we removed the background influence by applying the *normexp* method (Ritchie et al., 2007) with offset equals to 50. The top 20 spots with the highest pixel-level variability are highlighted in red plus symbols. Several of these spots have low average intensity (small estimates for $E\;(A_{tj})$) and a small difference between the intensities of the two channels (estimates for $E\;(M_{tj})$ close to zero), but they are not the majority. The differences between the proposed estimators, defined in Eq. (3) and (4), and the conventional estimators, defined in Eq. (5) and (6), are shown in Figures 1C, D. These differences are due to the distinct parts between their respective formulas. When computing the ${\tilde{M}}_{j}$ estimates, the ratio of the pixel-level variability to the squared expected value in the test channel appears in Eq. (3) with an opposite sign to the same term in the reference channel. Thus, positive and negative differences between the estimates for $E\;(M_{tj})$ may occur if such terms do not cancel each other out. **Figure 1C** shows the *ilde* ${\tilde{M}}_{t}$ estimates were smaller than the ${\hat{M}}_{t}$ estimates for the genes with highest pixel-level variance, indicating a larger variance in their test channels. Figure 1D shows some ${\tilde{A}}_{t}$ estimates were smaller than the ${\hat{A}}_{t}$ estimates. The reduction is explained by the fact that the additional terms in Eq. (4) are negative for any positive pixel-level variability in any channel.

#### Estimators for the Variances of the *M_t_* and *A_t_* Values

Since we have also available the sample covariance between *R_tj_* and *G_tj_*, denoted by $\hat{\sigma }(R_{t},G_{t})$, we applied the multivariate delta method for deriving estimators for the variances of the *M_tj_* and *A_tj_*. We calculated the variance of the first order Taylor’s expansion of the functions (1) and (2) that define, respectively, *M_tj_* and *A_tj_*, as shown in Appendix 5. The variance estimators for *M_tj_* and *A_tj_*, for pixels *j* within spot *t* are:

(7) $${\hat{\sigma }}^{2}(M_{t})≐\frac{1}{\ln^{2}(2)}\left(\frac{{\hat{\sigma }}^{2}(R_{t})}{{\bar{R}}_{tc}^{2}}+\frac{{\hat{\sigma }}^{2}(G_{t})}{{\bar{G}}_{tc}^{2}}-2\frac{\hat{\sigma }(R_{t},G_{t})}{{\bar{R}}_{tc}{\bar{G}}_{tc}}\right),$$

(8) $${\hat{\sigma }}^{2}(A_{t})≐\frac{1}{4\;\ln^{2}(2)}\left(\frac{{\hat{\sigma }}^{2}(R_{t})}{{\bar{R}}_{tc}^{2}}+\frac{{\hat{\sigma }}^{2}(G_{t})}{{\bar{G}}_{tc}^{2}}-2\frac{\hat{\sigma }(R_{t},G_{t})}{{\bar{R}}_{tc}{\bar{G}}_{tc}}\right).$$

The variances of *M_tj_* and represent pixel-level uncertainties of the th spot. They can be used, for instance, for assessing the quality of the th spot or for constructing confidence intervals for the parameters $E(M_{tj})$ and $E\;(A_{tj})$.

### Optimal Selection of the LOWESS Parameters

To simplify the notation, we will denote the estimates for $E\;(M_{tj})$ and $E(A_{tj})$, independently of the estimation method used, by, respectively, *M_t_* and *A_t_* values.

It is necessary to remove from these *M_tj_* intensity values the dependent dye-specific biases and other systematic errors by using some within-slide normalization method.

In the LOWESS within-slide normalization method, one estimates for each microarray slide a smoothing function $\hat{\mu }$ that maps each *A_t_* observed value to a smoothed *M_t_* value, $\hat{\mu }(A_{t})$. Since $\hat{\mu }(A_{t})$ is considered an estimate of a dye-dependent bias, it must be subtracted from the corresponding observed *M_t_* value to obtain a residual value representing, presumably, the biologically relevant gene expression level.

An appropriate LOWESS estimation depends on the choice of its parameters. According to loader (Loader, 1999), the weight function and the number of iterations of the robustness algorithm are not critical parameters. Cleveland (Cleveland, 1979) comments that good choices for these parameters are, respectively, the tricube function and three iterations. However, the degree of the local polynomials and the smoothing parameter *f*, which, in the nearest neighbor method, is a number between and indicating the proportion of data used in each local fit, affects the bias and the variance of the fit.

Specifically, the higher the degree of the local polynomial (related to the complexity of the model), the lower the bias of the fit (probably, fitting the data very well). However, the additional parameters of this more complex model increase the variance of the fitted values, yielding a poor generalization ability (i.e., the model will have a large error). Thus, to avoid unstable LOWESS estimates, several references as (Loader, 1999; Yang et al., 2001; Dudoit et al., 2002; Smyth and Speed, 2003) recommend using local polynomials of degree one, mainly in the presence of sparsity, as is the case of microarray data.

The effects of the smoothing parameter *f* on the bias and variance of the fit are opposite to those of the degree of the local polynomials. Since the *f* parameter indicates the number of observations that will be used in the local polynomial estimation, when *f* value is large, a simple polynomial may not fit well to all observations in the neighborhood, distorting or ignoring essential features. In other words, the estimation of the smoothing function can be significantly biased. On the other hand, when a low *f* value is chosen, the number of observations may be insufficient to capture the general behavior of the data, resulting in a very noisy (large variance) fitness function.

In the next section, we propose a method for selecting a value for the *f* parameter, focusing on microarray data normalization. Our method takes into account the intrinsic characteristics of the bias and variance of the fit as well as of gene expression data.

#### Lowess Smoothing Parameter Selection

For microarray data normalization, the ideal LOWESS fitted curve captures only trends and effects from systematic errors, retaining all biological variation. However, it critically depends on the choice of the *f* parameter value.

**Figure 2:**
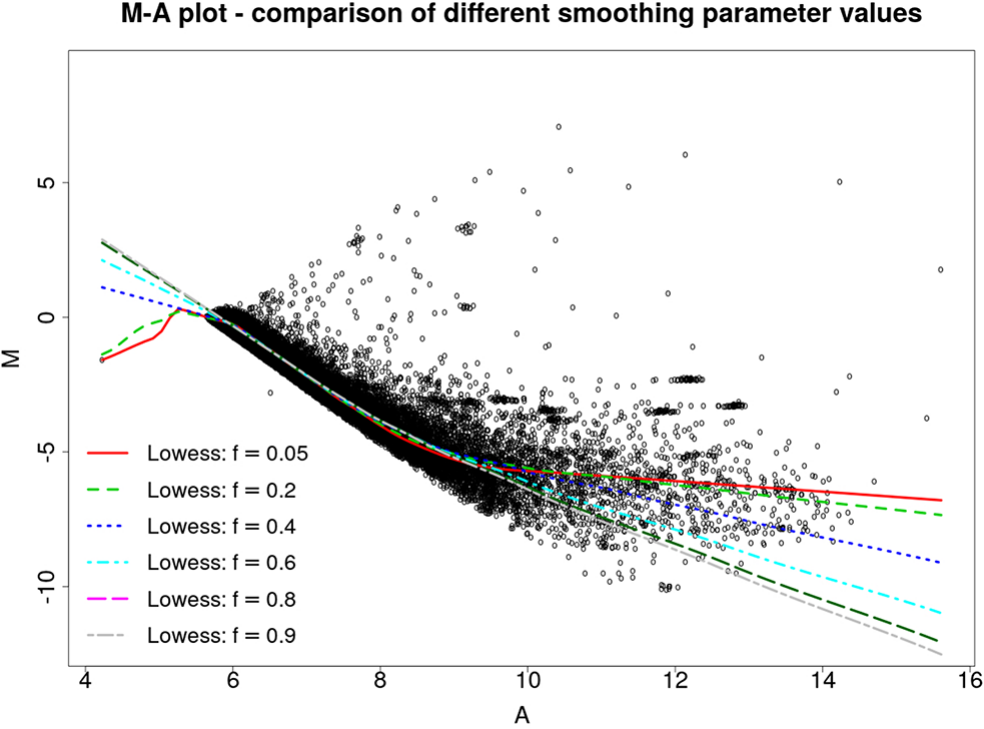
MA plot for the slide 251485069395_1.4, with *M_t_* and *A_t_* values estimated by the proposed method and LOWESS fits yielded by *f* values ranging from 0.05 to 0.9.

Figure 2 illustrates the MA plot of the microarray slide shown in Figure 1B, with different LOWESS fits yielded by *f* values varying from 0.05 to 0.9. The improved estimation method was used to obtain the *M_t_* and *A_t_* values, that is, the ${\tilde{M}}_{t}$ and ${\tilde{A}}_{t}$ estimates.

The quality of a LOWESS estimator can be assessed by the MSE, which measures how close the estimator $\hat{\mu }$ is of the true mean function μ :

$$MSE(\hat{\mu })=E[{\left(\mu -\hat{\mu }\right)}^{2}].$$

Since the real curve μ is unknown, we need a criterion to evaluate the MSE. Under the assumption of heteroskedasticity, Cleveland and Devlin (Cleveland and Devlin, 1988) propose the Mallows’ Cp criterion for local fitting that can be used as as MSE estimator. In the presence of heteroskedasticity, as usual for microarray data, the heteroskedasticity-robust Cp (HRCp) criterion, proposed by Liu and Okui (Liu and Okui, 2013), may be a more appropriate MSE estimator. We detail this MSE estimator next.

Considering ${\left\{(A_{t},M_{t})\right\}}_{t=1}^{T}$ within-slide data points, the evaluation of the LOWESS smoothing function on any point is given by a linear combination of the observed points, whose weights $\{{\left(l_{t}(A)\right\}}_{t=1}^{T}$ are assigned according to the distance of *A* to the *A_t_* observed points:

$$\hat{\mu }(A)=\overset{T}{\underset{t=1}{\sum }}l_{t}(A)M_{t}.$$

Consider the *T* × *T* matrix ***L*** which maps the observed to the fitted values:

$$\left(\begin{array}{c} \hat{\mu }(A_{1})\\ \vdots \\ \hat{\mu }(A_{T}) \end{array}\right)=LM=\left(\begin{array}{c} l_{1}(A_{1})\;\ldots \;l_{T}(A_{1})\\ \vdots \\ l_{1}(A_{T})\;\ldots \;l_{T}(A_{T}) \end{array}\right)\left(\begin{array}{c} M_{1}\\ \vdots \\ M_{T} \end{array}\right).$$

Two commons definitions of the effective degrees of freedom of $\hat{\mu }$ are: (1) $v_{1}≐tr\;(L)$ and (2) $v_{2}≐tr\;(L^{\prime }L)$, where tr stands for the trace operator.

Supposing that the variance of *M_t_*, across *T* spots of a microarray slide, is constant and equals to σ^2^, the Mallows’ Cp for local fitting is defined as:

$$Cp(\hat{\mu })=\frac{1}{\sigma^{2}}\overset{T}{\underset{t=1}{\sum }}{\left(M_{t}-\hat{\mu }(A_{t})\right)}^{2}-T+2v_{1}.$$

Cleveland et al. (1988) shows that σ^2^ can be estimated as follows:

$${\hat{\sigma }}^{2}≐\frac{\Sigma_{t=1}^{T}{\left[M_{t}-\hat{\mu }(A_{t})\right]}^{2}}{n+v_{2}-2v_{1}}.$$

When heteroskedasticity is present, Mallows’ Cp criterion is not an appropriate MSE estimator. Considering the *T* × *T* diagonal matrix Σ, whose th diagonal element is given by a non-homogeneous variance $\sigma_{t}^{2}$ of *M_t_*, a robust MSE estimation can be achieved by using the HRCp criterion, defined as:

$$HRCp(\hat{\mu })=\overset{T}{\underset{t=1}{\sum }}{\left(M_{t}-\hat{\mu }(A_{t})\right)}^{2}+2\text{tr }(\Sigma L).$$

According to Loader (1999), $\sigma_{t}^{2}$ can be estimated locally by calculating the error variance (the residual sum of squares divided by the corresponding degrees of freedom) of a nearly unbiased LOWESS fit, which can be yielded using a very small value for the smoothing parameter (e.g., *f* = 0.1. Since the local variance estimates can be very noisy, it may be appropriate to smooth them using a gamma kernel.

Several authors suggest to choose the *f* value which minimizes a measure of error of the LOWESS fit, such as the MSE criterion (Berger et al., 2004; Futschik and Crompton, 2004a; Lee et al., 2008). However, other authors (Mallows, 1973; Cleveland and Devlin, 1988; Loader, 1999) argue that a selection based only on minimizing the MSE criterion is a poor procedure since it ignores the intrinsic information of the bias and variance of the fit. Therefore, following their suggestion, we propose a method based on a graphical tool called M-plot. It is a graph of the MSE estimate as a function of the effective degrees of freedom of the fit.

**Figure 3:**
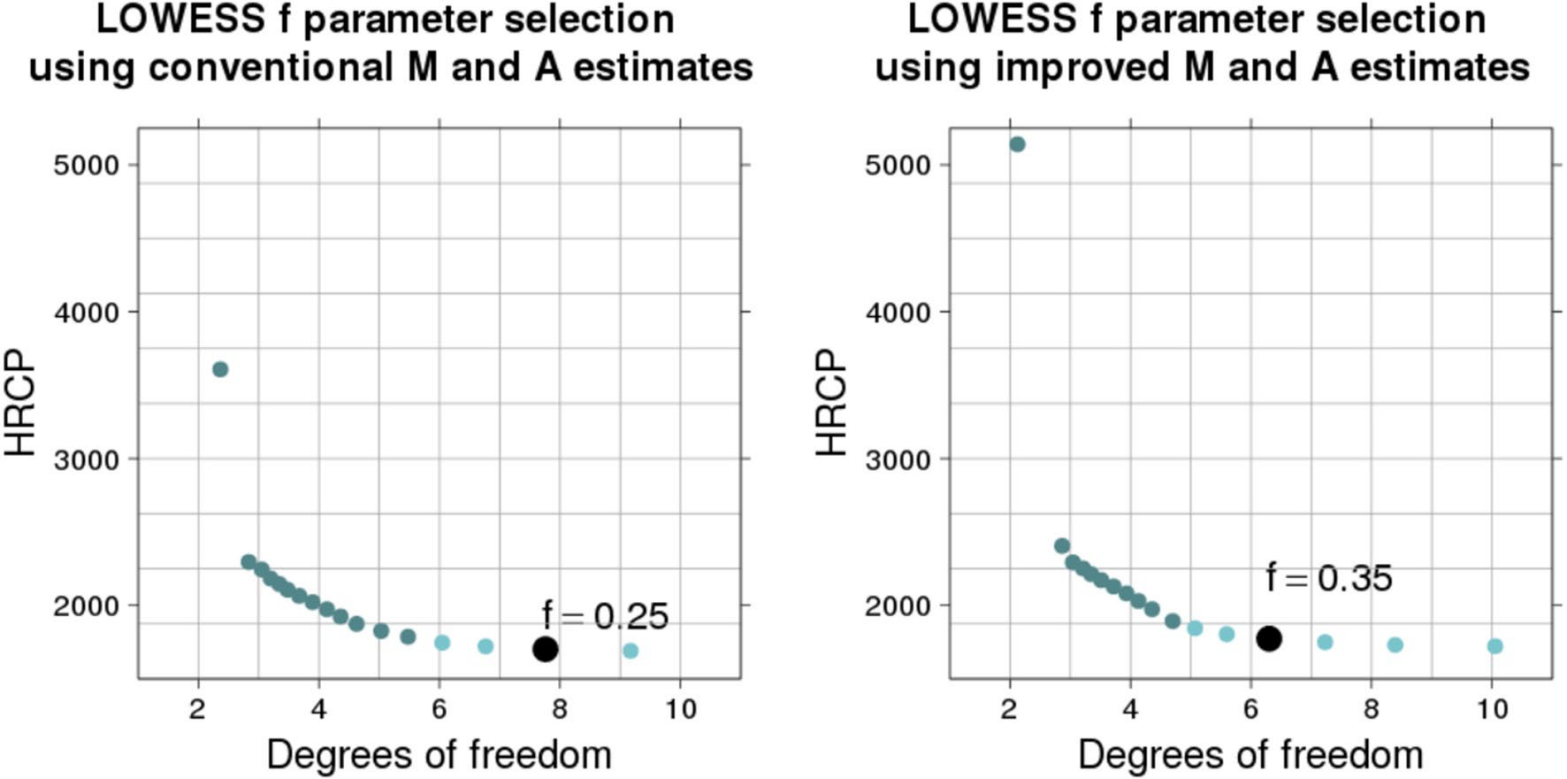
Selection of the LOWESS *f* parameter by using HRCp criterion. The M-plots illustrate the selection process for a particular microarray slide (ID: 251485069395_1.4). The flattening region is represented by the light-colored dots and the selected *f* value by the biggest dot. The LOWESS fits were yielded using values ranging from 1 to 0.2 (from lowest to highest degree of freedom).

M-plots illustrating the *f* parameter selection method for a typical microarray slide (ID 251485069395_1.4) are shown in Figure 3. Dots show MSE estimates (by HRCp criterion) and respective degrees of freedom (by *v*_2_ definition) of LOWESS fits (on the ${\hat{M}}_{t}$ and ${\hat{A}}_{t}$ estimates, in the first M-plot, and on the ${\tilde{M}}_{t}$ and ${\tilde{A}}_{t}$, in the second M-plot) obtained with *f* parameter varying from to 0.2 We fixed the other LOWESS parameters (local polynomials of degree one, tricube weight function, and three iterations) so that the M-plot curve shows only the effect of the *f* parameter on the bias–variance compromise. Large *f* values tend to yield simple fits (with fewer degrees of freedom), which have a small variance, but a large bias. On the other hand, minimal *f* values tend to yield complex fits (with many degrees of freedom), which have a small bias, but a large variance.

For the microarray slide in Figure 3, a selection method based only on the minimization of the MSE curve would choose the smallest evaluated *f* value (0.2). However, any *f* value within the flattening region near to the minimum (the region with light-colored dots) is a good choice, in the sense that it yields a low-MSE fit (Cleveland and Devlin, 1988; Loader, 1999). Depending on the type of application, we can choose between one value which yields a low-bias fit (with more degrees of freedom) or a low-variance fit (with fewer degrees of freedom). Since we want to estimate a natural phenomenon behavior, we propose to select from the flattening region the *f* value which yields the simplest LOWESS fit (the one with fewest effective degrees of freedom). The biggest dot in each M-plot indicates the selected *f* value. The detection of the flattening region is made by searching points for which the derivative of the MSE curve is small. We check for each sequence of three points near the minimum whether the difference between the MSE values is small. If so, these points are considered as belonging to the flattening region.

The *f* parameter selection method can be summarized in the following discrete and constrained optimization problem. Consider a sequence of *l* different values for *f*, {*f*_1_, *f*_2_, ... , *f_l_*}, and denoted by ${\hat{\mu }}_{f_{k}}$, the LOWESS fit yielded by using the value *f_k_* for the *f* parameter. Also, let:

$$\begin{array}{l} ℱ=\{{\hat{\mu }}_{f_{k}};f_{k}\in \{f_{1},f_{2},\ldots ,f_{l}\},\;f_{k+1}<f_{k},\;for\;k=1,\ldots ,l-1\};\\ f_{min}=\underset{f_{k}}{\arg\;\min\;}\;HRCp\;({\hat{\mu }}_{f_{k}}),\;such\;that\;{\hat{\mu }}_{f_{k}}\in ℱ;\\ f_{max}=\underset{f_{k}}{\arg\;\max\;}\;HRCp\;({\hat{\mu }}_{f_{k}}),\;such\;that\;{\hat{\mu }}_{f_{k}}\in ℱ;\;and\\ {∆}_{MSE}=0.05(HRCp\;({\hat{\mu }}_{f_{\max}})-HRCp\;({\hat{\mu }}_{f_{\min}})). \end{array}$$

Since *v*_2_ function provides the effective degrees of freedom of a given fit, the selected *f* value is the solution f^*^, if it exists, of the following problem:

$$\begin{array}{l} f^{*}≐\underset{f_{k}}{\arg\;\min\;}\;\nu_{2}({\hat{\mu }}_{f_{k}})\\ \text{subject to}:\\ {\hat{\mu }}_{f_{k}}\in ℱ;\\ \text{HRCp }({\hat{\mu }}_{f_{k}})\;\leq \text{ HRCp }({\hat{\mu }}_{f_{min}})\;+\;{∆}_{MSE},\text{ for }k=1,2;\\ \text{HRCp }({\hat{\mu }}_{f_{k-2}})\;\leq \text{ HRCp }({\hat{\mu }}_{f_{min}})\;+\;{∆}_{MSE},\text{ for }k=3,\ldots ,l;\\ |\text{HRCp }({\hat{\mu }}_{f_{k}})\;-\text{ HRCp }({\hat{\mu }}_{f_{k-1}})|\;<\;{∆}_{MSE},\text{ for }k=2,\ldots ,l;\text{ and}\\ |\text{HRCp }({\hat{\mu }}_{f_{k}})\;-\text{ HRCp }({\hat{\mu }}_{f_{k-2}})|\;<\;{∆}_{MSE},\text{ for }k=3,\ldots ,l. \end{array}$$

If the minimum of the M-plot curve is far away of the point corresponding to the second lowest MSE estimate, the previous problem has no solution. In that case, the *f* value that yields the fit with lowest MSE estimate is selected. Specifically, the *f* parameter value is selected by solving the following problem:

$$f^{*}≐\underset{f_{k}}{\arg\;\min\;}\text{ HRCp }({\hat{\mu }}_{f_{k}}),\text{ such that }{\hat{\mu }}_{f_{k}}\in ℱ.$$

## Application on Intestinal Metaplasia Data

To investigate the effects of the proposed methods, we preprocessed the data described in the section *Intestinal Metaplasia Database* by using all discussed methods and compared the identified differentially expressed genes.

First, we applied the *normexp* method with offset value of for removing the background influence. Then, we compute the *M_t_* and *A_t_* values both by the conventional estimation methods, defined in Eq. (5) and (6), and by the proposed estimation methods, defined in Eq. (3) and (4). The LOWESS within-slide normalization was carried out as discussed in the section *Optimal Selection of the LOWESS Parameters*. For comparison purpose, the *f* smoothing parameter was selected both by the OLIN method (considered by us as a conventional approach) and by the proposed method, discussed in the section *LOWESS Smoothing Parameter Selection*. Since data from all microarray slides present overdispersion, we used the HRCp criterion as cost function of our selection method.

Therefore, the following four preprocessing procedures were applied separately to the original data:

1. Conventional estimation for *M_t_* and *A_t_* and LOWESS within-slide normalization using *f* parameter selected by OLIN;
2. Improved estimation of *M_t_* and *A_t_* and LOWESS within-slide normalization using *f* parameter selected by OLIN;
3. Conventional estimation of *M_t_* and *A_t_* and LOWESS within-slide normalization using *f* parameter selected by the proposed method;
4. Improved estimation of *M_t_* and *A_t_* and LOWESS within-slide normalization using parameter selected by the proposed method.

**Figure 4:**
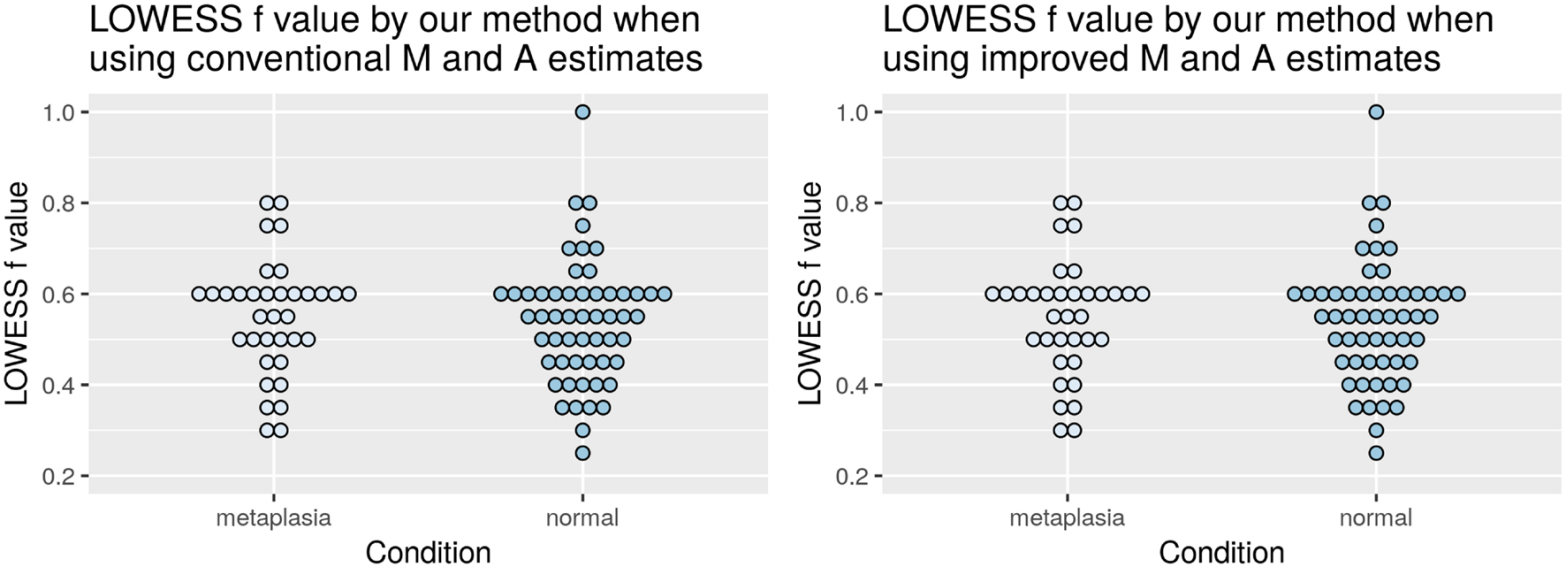
Distribution of the selected *f* values by normal and metaplasia intestinal conditions when the *M_t_* and *A_t_* values are estimated by using the conventional (left) and the proposed (right) method.

**Figure 4** shows the distribution of the optimal values for the LOWESS *f* parameter, according to the proposed selection method with HRCp criterion, for the entire database, separated by normal and intestinal metaplasia conditions (both, hybridized against a pool of normal tissues). In the first plot, the LOWESS curve was fitted on the ${\hat{M}}_{t}$ and ${\hat{A}}_{t}$ estimates and, in the second plot, on the ${\tilde{M}}_{t}$ and ${\tilde{A}}_{t}$ estimates. The average of the selected *f* values was close to 0.5.

As expected from a method that neither takes into account heteroskedasticity of the data nor attempts to make a good balance between bias and variance, the OLIN method selected the smallest evaluated value (0.2) for most of the slides. Same results were obtained when the *M_t_* and *A_t_* values were estimated by the conventional and by the proposed estimator. Such behavior has been reported in the literature, implying that the optimal *f* values according OLIN are usually close to the default one (Chiogna et al., 2009).

After preprocessing the data, a two-sample t-test assuming unequal variance was performed for each spotted gene to determine whether its expression is statistically different between gastric tissues in normal and intestinal metaplasia groups. However, since we are interested in directly assessing the impact of each proposed method on the t-statistics and p-values rather than making inference about differential expression, the comparative study was performed before applying a multiple testing correction.

### Comparison of the Results

**Figure 5:**
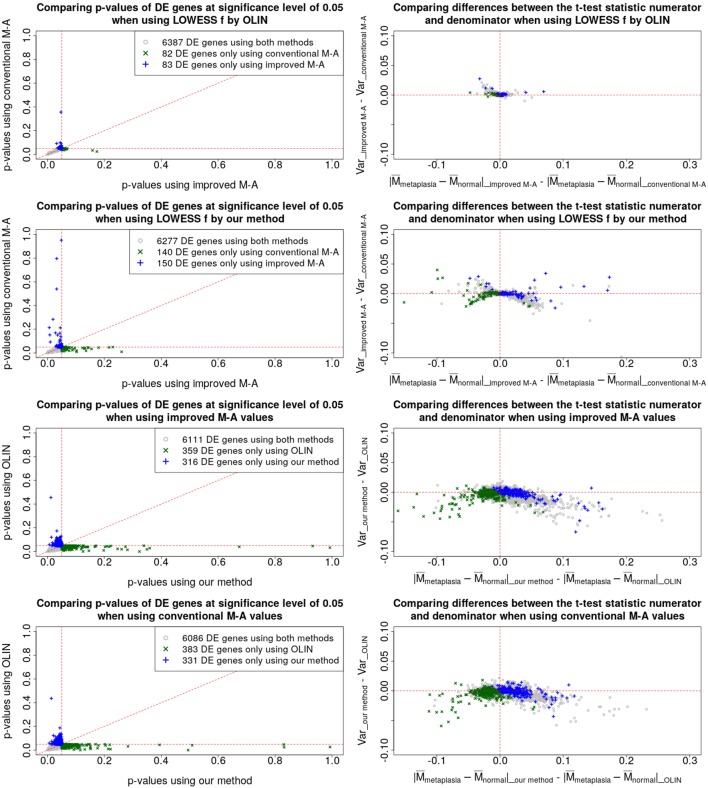
Pairwise comparison between the proposed and the conventional methods. Left-column plots compare the FDR-corrected p-values, and the right-column plots compare the difference between the absolute values of the numerators with the difference between the denominators of the t-test statistic.

Results of a pairwise comparison among the p-values and t-statistics obtained by the four preprocessing methods are shown in **Figure 5**. In the left-column plots, we compare the p-values and, in the right-column plots, we show the changes in the difference between the group means (the absolute value of the t-statistic numerator) and in the within-group variability (the denominator of the t-statistic). Only genes with p-value less than 5% were considered.

The left-column plots show that most of the points are distributed around the 45-degree line. Thus, the p-values and, consequently, the total number of differentially expressed genes, even at a lower significance level, were similar among the four methods.

The first- and second-row plots show how p-values and t-statistics were affected by estimating the *M_t_* and *A_t_* values with the proposed method, which takes into account the pixel-level uncertainties. The genes represented by blue plus signs were identified as differentially expressed only when using the proposed estimator for the *M_t_* and *A_t_* values.

The genes represented by green crosses were identified as differentially expressed only when using the conventional estimator for the *M_t_* and *A_t_* values.

When the LOWESS *f* parameter is selected by OLIN (first-row plots), it is clear that the within-group variability decreases when using the proposed estimators for the *M_t_* and *A_t_* values. When the LOWESS parameter is selected by our method (second-row plots), there is still a reduction in the within-group variability. However, this impact is less clear because of the variability introduced when the LOWESS *f* parameter is selected by our method.

The third- and fourth-row plots compare p-values and t-statistics obtained by OLIN and the proposed approach for selecting the LOWESS *f* parameter. The genes represented by blue plus signs were identified as differentially expressed only when *f* was selected by the proposed method. The genes represented by green crosses were identified as differentially expressed only when selecting *f* by OLIN. It is clear that, for most genes, both within-group variabilities increased, implying that the normalization procedure was more conservative, and thus, more potentially relevant information is retained. In addition, for many genes, the increase in the within-group variability was counterbalanced by an increase in the distance between the groups. Such effect is even most pronounced when the proposed estimator for the *M_t_* and *A_t_* values are used. Thus, their respective p-values reduced enough to consider them as differentially expressed genes.

**Figure 6:**
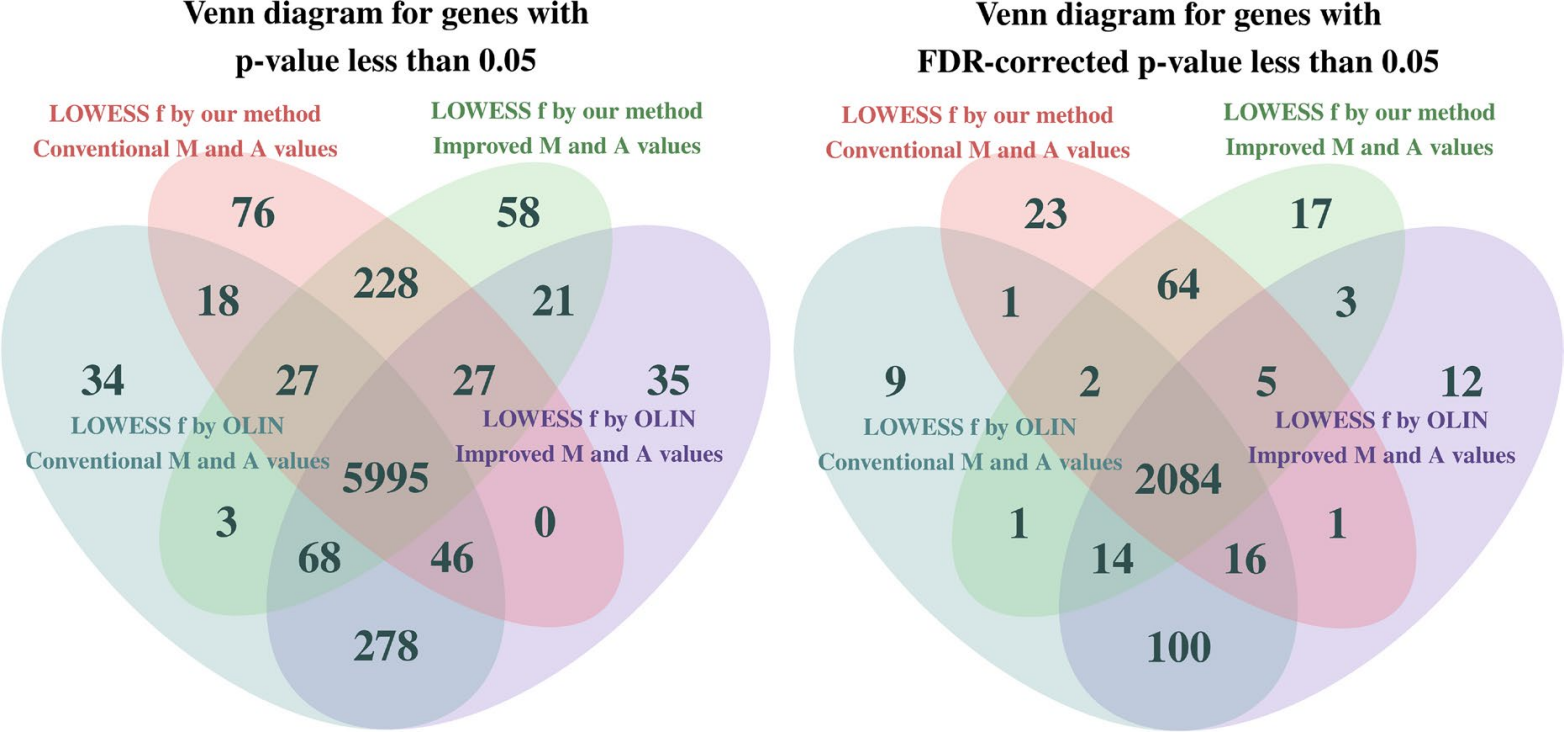
Venn diagram illustrating the total number of differentially expressed genes identified in each variant of the database at a significance level of 5%. On the left, p-values were not corrected for multiple tests, while on the right, p-values were adjusted by the false discovery rate (FDR) correction.

The diagrams in **Figure 6** show a comparison of the methods with respect to the total number of genes with p-value less than 5%. On the left, the p-values were not corrected for multiple tests, while on the right, the p-values were adjusted by the false discovery rate (FDR) correction (Benjamini and Hochberg, 1995).

Note that the four methodologies are quite different in terms of which genes were identified as differentially expressed. As a consequence of the more conservative (milder) noise reduction performed in the LOWESS within-slide normalization procedure with *f* parameter selected by our method, fewer genes are identified as differentially expressed. However, regardless of the normalization method, more genes could be identified as differentially expressed when the *M_t_* and *A_t_* values were estimated by the proposed estimation method that incorporates pixel-level variability. Given that both proposed methods make the analysis more robust by incorporating and preserving information neglected by the conventional methods, we can argue that they are contributing to the reduction of both false-positive and false-negative rates.

### Validation Analysis

**Figure 7:**
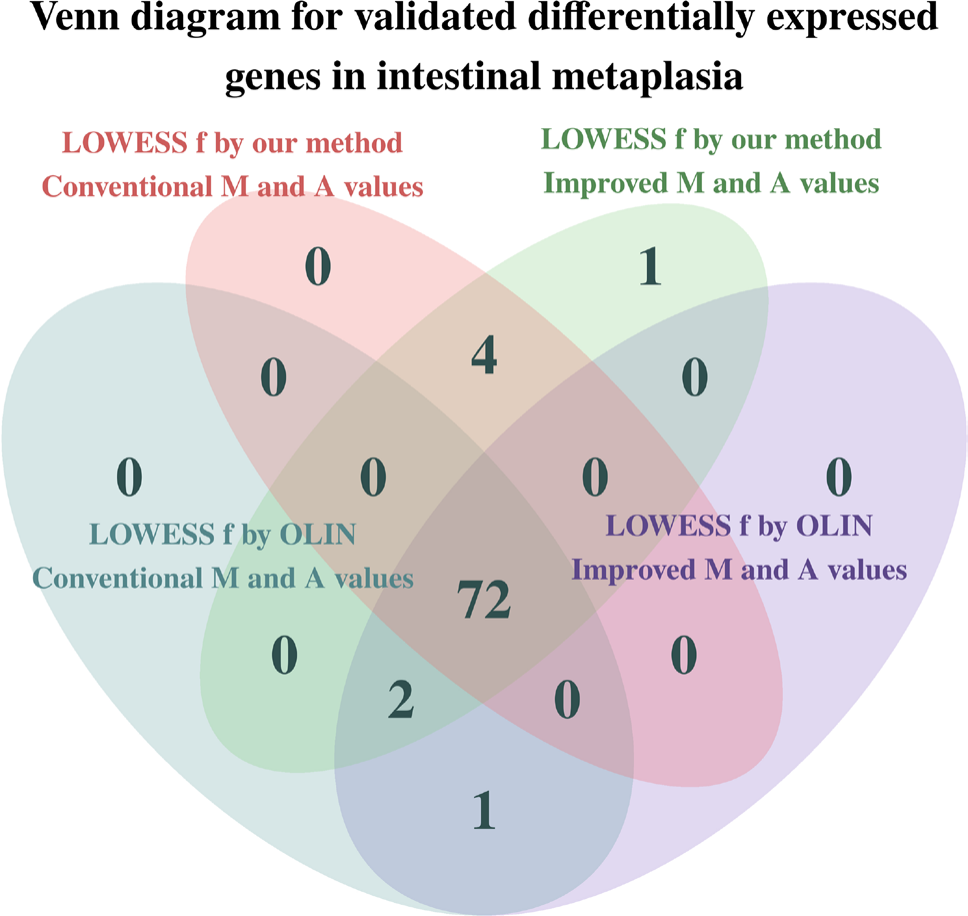
Venn diagram for the total number of genes already identified as differentially expressed in intestinal metaplasia according to the literature. Inferences were made at a significance level of 5%.

To check the consistency of our analysis, we compared our results with those reported in the literature. Out of the genes which are associated with intestinal metaplasia according to the Gene Expression Omnibus platform (Edgar et al., 2002) of the NCBI (National Center for Biotechnology Information) website, 80 spotted genes (corresponding to 63 unique genes) have p-value (before FDR correction) less than 5%, and 35 spotted genes (corresponding to 29 unique genes) have p-value (after FDR correction) less than 5%. These findings are summarized respectively in **Tables 1**, **2**. In addition, **Figure 7** compares the total number of validated genes identified by each method with p-value less than 5% (before FDR correction).

**Table 1:** Genes reported in the literature as associated with intestinal metaplasia of the stomach that were identified as differentially expressed in our analysis at a significance level of 5% (after FDR correction).

Gene	Improved estimation for the and values						Conventional estimation for the *M_t_* and *A_t_* values					
	*f* by our method			*f* by OLIN			*f* by our method			*f* by OLIN		
	p	adj. p	FC	p	adj. p	FC	p	adj. p	FC	p	adj. p	FC
CLND3	2.70 × 10^−12^	4.28 × 10^−8^	2.86	1.84 × 10^−12^	2.32 × 10^−8^	2.74	2.77×10^−12^	4.01 × 10^−8^	2.86	1.87 × 10^−12^	2.33 × 10^−8^	2.74
CLND3	2.23 × 10^−5^	1.35 × 10^−3^	0.59	1.63 × 10^−5^	1.07 × 10^−3^	0.60	2.23 × 10^−5^	1.35 × 10^−3^	0.59	1.55 × 10^−5^	1.04 × 10^−3^	0.60
MUC2	3.51 × 10^−11^	1.32 × 10^−7^	1.73	3.14 × 10^−11^	1.06 × 10^−7^	1.71	3.21 × 10^−11^	1.21 × 10^−7^	1.73	3.06 × 10^−11^	1.04 × 10^−7^	1.71
MUC2	1.90 × 10^−4^	6.56×10^−3^	0.24	2.14 × 10^−4^	7.19 × 10^−3^	0.24	1.96 × 10^−4^	6.69 × 10^−3^	0.24	2.35 × 10^−4^	7.74 × 10^−3^	0.23
CDX1	4.22 × 10^−10^	6.05 × 10^−7^	2.15	4.53 × 10^−10^	6.74 × 10^−7^	2.13	4.03 × 10^−7^	5.94 × 10^−7^	2.16	4.40 × 10^−10^	6.98 × 10^−7^	2.14
ANPEP	4.28 × 10^−10^	6.05 × 10^−7^	3.14	5.31 × 10^−10^	7.19 × 10^−7^	3.08	4.37 × 10^−10^	6.17 × 10^−7^	3.13	5.19 × 10^−10^	7.03 × 10^−7^	3.07
CLCA1	2.55 × 10^−9^	1.69 × 10^−6^	3.75	7.18 × 10^−10^	8.49 × 10^−7^	3.85	2.71 × 10^−9^	1.70 × 10^−6^	3.75	7.15 × 10^−10^	8.93 × 10^−7^	3.85
DMBT1	2.79 × 10^−9^	1.75 × 10^−6^	3.39	4.22 × 10^−9^	2.43 × 10^−6^	3.26	2.77 × 10^−9^	1.71 × 10^−6^	3.39	3.98 × 10^−9^	2.33 × 10^−6^	3.26
GUCY2C	3.07 × 10^−9^	1.86 × 10^−6^	2.31	9.58 × 10^−9^	4.07 × 10^−6^	2.20	3.10 × 10^−9^	1.84 × 10^−6^	2.31	9.70 × 10^−9^	4.06 × 10^−6^	2.19
CLDN7	3.78 × 10^−9^	2.17 × 10^−6^	2.37	2.21 × 10^−9^	1.56 × 10^−6^	2.23	1.24 × 10^−9^	1.13 × 10^−6^	2.27	2.30 × 10^−9^	1.59 × 10^−6^	2.22
CDH17	4.21 × 10^−9^	2.27 × 10^−6^	2.69	4.83 × 10^−9^	2.64 × 10^−6^	2.65	4.16 × 10^−9^	2.24 × 10^−6^	2.69	4.73 × 10^−9^	2.59 × 10^−6^	2.65
CDX2	5.67 × 10^−9^	2.80 × 10^−6^	1.01	7.29 × 10^−9^	3.40 × 10^−6^	1.00	6.00 × 10^−9^	2.82 × 10^−6^	1.01	7.67 × 10^−9^	3.51 × 10^−6^	1.00
DEFA5	1.17 × 10^−7^	2.48 × 10^−5^	3.33	1.17 × 10^−7^	2.45 × 10^−5^	3.29	1.18 × 10^−7^	2.46 × 10^−5^	3.32	1.17 × 10^−7^	2.43 × 10^−5^	3.28
VDR	2.82 × 10^−7^	4.94 × 10^−5^	1.15	1.61 × 10^−7^	3.23 × 10^−5^	1.12	2.60 × 10^−7^	4.64 × 10^−5^	1.15	1.57 × 10^−7^	3.17 × 10^−5^	1.12
ISX	5.26 × 10^−7^	8.04 × 10^−5^	1.33	5.57 × 10^−7^	8.25 × 10^−5^	1.32	5.37 × 10^−7^	8.06 × 10^−5^	1.33	5.83 × 10^−7^	8.03 × 10^−5^	1.31
CLDN4	1.15 × 10^−6^	1.43 × 10^−4^	1.20	1.33 × 10^−6^	1.62 × 10^−4^	1.19	1.15 × 10^−6^	1.40 × 10^−4^	1.19	1.33 × 10^−6^	1.60 × 10^−4^	1.18
ACSL5	2.44 × 10^−6^	2.49 × 10^−4^	1.45	2.29 × 10^−6^	2.42 × 10^−4^	1.45	2.17 × 10^−6^	2.26 × 10^−4^	1.46	2.16 × 10^−6^	2.30 × 10^−4^	1.45
REG4	3.24 × 10^−6^	3.06 × 10^−4^	2.50	3.53 × 10^−6^	3.35 × 10^−4^	2.45	3.21 × 10^−6^	3.02 × 10^−4^	2.50	3.49 × 10^−6^	3.31 × 10^−4^	2.45
REG4	3.62 × 10^−4^	1.08 × 10^−2^	1.28	1.41 × 10^−3^	2.84 × 10^−2^	1.11	3.57 × 10^−4^	1.06 × 10^−2^	1.28	1.35 × 10^−3^	2.76 × 10^−2^	1.11
RUNX1	1.11 × 10^−5^	7.87 × 10^−4^	−0.56	6.91 × 10^−6^	5.50 × 10^−4^	−0.57	1.01 × 10^−5^	7.16 × 10^−4^	−0.55	7.59 × 10^−6^	5.93 × 10^−4^	−0.57
FOXA2	1.12 × 10^−5^	7.90 × 10^−4^	−1.13	9.18 × 10^−6^	6.75 × 10^−4^	−1.14	1.10 × 10^−5^	7.72 × 10^−4^	−1.13	9.51 × 10^−6^	6.96 × 10^−4^	−1.13
FOXA2	1.67 × 10^−4^	5.93 × 10^−3^	−0.86	2.12 × 10^−4^	7.16 × 10^−3^	−0.85	1.73 × 10^−4^	6.10 × 10^−3^	−0.86	2.22 × 10^−4^	7.42 × 10^−3^	−0.85
FOXA2	7.25 × 10^−3^	**8.39** × **10^−2^**	−0.61	8.20 × 10^−3^	**9.01** × **10^−2^**	−0.60	7.71 × 10^−3^	**8.67** × **10^−2^**	−0.61	8.13 × 10^−3^	**9.03** × **10^−2^**	−0.60
SOX2	1.62 × 10^−5^	1.05 × 10^−3^	−0.87	1.44 × 10^−5^	9.73 × 10^−4^	−0.87	1.56 × 10^−5^	1.01 × 10^−3^	−0.87	1.38 × 10^−5^	9.41 × 10^−4^	−0.87
SOX2	1.48 × 10^−4^	5.50 × 10^−3^	−0.77	3.23 × 10^−4^	9.87 × 10^−3^	−0.74	1.55 × 10^−4^	5.62 × 10^−3^	−0.76	3.26 × 10^−4^	1.00 × 10^−2^	−0.73
SERPINB5	2.42 × 10^−5^	1.44 × 10^−3^	1.04	2.55 × 10^−5^	1.51 × 10^−3^	1.03	2.46 × 10^−5^	1.45 × 10^−3^	1.05	2.67 × 10^−5^	1.58 × 10^−2^	1.03
SERPINB5	1.15 × 10^−4^	4.59 × 10^−3^	0.64	1.18 × 10^−4^	4.65 × 10^−3^	0.64	1.13 × 10^−4^	4.49 × 10^−3^	0.64	1.14 × 10^−4^	4.52 × 10^−3^	0.64
SERPINB5	1.73 × 10^−2^	**1.42** × **10^−1^**	0.11	1.18 × 10^−2^	**1.14** × **10^−1^**	0.12	1.66 × 10^−2^	**1.39** × **10^−1^**	0.11	1.22 × 10^−2^	**1.16** × **10^−1^**	0.11
FAS	6.35 × 10^−5^	2.95 × 10^−3^	0.41	6.54 × 10^−5^	3.02 × 10^−3^	0.41	6.46 × 10^−5^	2.97 × 10^−3^	0.41	6.93 × 10^−5^	3.12 × 10^−3^	0.41
CDHI	2.13 × 10^−4^	7.14 × 10^−3^	0.62	1.97 × 10^−4^	6.74 × 10^−3^	0.60	1.88 × 10^−4^	6.50 × 10^−3^	0.62	2.05 × 10^−4^	6.94 × 10^−3^	0.60
EMPI	6.05 × 10^−4^	1.57 × 10^−2^	0.94	6.45 × 10^−4^	1.65 × 10^−2^	0.90	5.77 × 10^−4^	1.51 × 10^−2^	0.94	6.61 × 10^−4^	1.68 × 10^−2^	0.90
EMPI	7.22 × 10^−3^	**8.36** × **10^−2^**	0.37	5.94 × 10^−3^	**7.37** × **10^−2^**	038	7.02 × 10^−3^	**8.19** × **10^−2^**	0.37	5.95 × 10^−3^	**7.39** × **10^−2^**	0.37
FGFR2	7.50	1.86 × 10^−2^	−0.57	9.15 × 10^−4^	2.12 × 10^−2^	−0.58	7.44 × 10^−4^	1.83 × 10^−2^	−0.57	9.13 × 10^−4^	2.11 × 10^−2^	−0.57
FGFR2	8.37 × 10^−3^	**9.20** × **10^−2^**	−0.12	7.95 × 10^−3^	**8.85** × **10^−2^**	−0.12	9.07 × 10^−3^	**9.65** × **10^−2^**	−0.12	8.47 × 10^−3^	**9.23** × **10^−2^**	−0.12
PGC	9.29 × 10^−4^	2.15 × 10^−2^	−1.71	1.47 × 10^−3^	2.92 × 10^−2^	−1.45	7.65 × × 10^−4^	1.87 × 10^−2^	−1.64	1.46 × 10^−3^	2.91 × 10^−2^	−1.45
LRIG1	9.74 × 10^−4^	2.22 × 10^−2^	−0.67	4.82 × 10^−4^	1.34 × 10^−2^	−0.67	8.72 × 10^−4^	2.04 × 10^−2^	−0.66	5.07 × 10^−4^	1.39 × 10^−2^	−0.67
KRT20	1.05 × 10^−3^	2.32 × 10^−2^	1.49	1.18 × 10^−3^	2.52 × 10^−2^	1.46	1.02 × 10^−3^	2.26 × 10^−2^	1.49	1.17 × 10^−3^	2.50 × 10^−2^	1.46

Each column shows the p-value (p), the FDR-corrected p-value (adj. p), and the fold change (FC) obtained in a variant of the database. P-values greater than 5% are shown in bold type.

**Table 2:** Other genes reported in the literature as associated with intestinal metaplasia of the stomach that were identified as differentially expressed in our analysis at a significance level of 5% (without FDR correction).

Gene	Improved estimation for the *M_t_* and *A_t_* values						Conventional estimation for the *M_t_* and *A_t_* values					
	*f* by our method			*f* by OLIN			*f* by our method			*f* by OLIN		
	p	adj. p	FC	p	adj. p	FC	p	adj. p	FC	p	adj. p	FC
VEGFA	3.76 × 10^−3^	**5.52** × **10^−2^**	−0.76	4.16 × 10^−3^	**5.84** × **10^−2^**	−0.75	3.54 × 10^−3^	**5.28** × **10^−2^**	−0.76	4.21 × 10^−3^	× **10^−2^**	−0.75
VEGFA	4.03 × 10^−2^	**2.35** × **10^−1^**	−0.25	3.93 × 10^−2^	**2.29** × **10^−1^**	−0.25	4.65 × 10^−2^	**2.54** × **10^−1^**	−0.25	4.52 × 10^−2^	× **10^−1^**	−0.25
PPP1R1B	3.96 × 10^−3^	**5.70** × **10^−2^**	0.76	4.07 × 10^−3^	**5.76** × **10^−2^**	0.75	3.89 × 10^−3^	**5.60** × **10^−2^**	0.76	4.03 × 10^−3^	× **10^−2^**	0.75
MUC5AC	4.07 × 10^−3^	**5.79** × **10^−2^**	−1.08	3.54 × 10^−3^	**5.24** × **10^−2^**	−1.08	4.18 × 10^−3^	**5.87** × **10^−2^**	−1.07	3.58 × 10^−3^	× **10^−2^**	−1.08
MUC5AC	4.60 × 10^−3^	**6.30** × **10^−2^**	−0.83	4.51 × 10^−3^	**6.15** × **10^−2^**	−0.82	4.78 × 10^−3^	**6.40** × **10^−2^**	−0.82	4.50 × 10^−3^	× **10^−2^**	−0.82
CLDN18	4.78 × 10^−3^	**6.46** × **10^−2^**	−1.05	5.12 × 10^−3^	**6.69** × **10^−2^**	−1.03	4.83 × 10^−3^	**6.44** × **10^−2^**	−1.04	5.03 × 10^−3^	× **10^−2^**	−1.03
ASCC1	6.62 × 10^−3^	**7.90** × **10^−2^**	0.18	1.42 × 10^−2^	**1.27** × **10^−1^**	0.17	6.57 × 10^−3^	**7.85** × **10^−2^**	0.18	1.43 × 10^−2^	× **10^−1^**	0.17
FOXA3	6.85 × 10^−3^	**8.09** × **10^−2^**	−0.57	4.87 × 10^−3^	**6.47** × **10^−2^**	−0.57	6.98 × 10^−3^	**8.15** × **10^−2^**	−0.56	5.01 × 10^−3^	× **10^−2^**	−0.57
FOXA3	1.96 × 10^−2^	**1.54** × **10^−1^**	−0.53	2.05 × 10^−2^	**1.58** × **10^−1^**	−0.52	1.98 × 10^−2^	**1.54** × **10^−1^**	−0.53	1.98 × 10^−2^	× **10^−1^**	−0.52
GAST	8.99 × 10^−3^	**9.60** × **10^−2^**	−1.48	1.24 × 10^−2^	**1.17** × **10^−1^**	−1.31	9.15 × 10^−3^	**9.69** × **10^−2^**	−1.48	1.21 × 10^−2^	× **10^−1^**	−1.32
PIK3CA	1.02 × 10^−2^	**1.04** × **10^−1^**	−0.16	7.28 × 10^−3^	**8.42** × **10^−2^**	−0.17	9.62 × 10^−3^	**9.97** × **10^−2^**	−0.16	6.62 × 10^−3^	× **10^−2^**	−0.17
BHLHA15	1.04 × 10^−2^	**1.05** × **10^−1^**	−0.63	9.50 × 10^−3^	**9.93** × **10^−2^**	−0.63	1.11 × 10^−2^	**1.09** × **10^−1^**	−0.62	9.79 × 10^−3^	× **10^−1^**	−0.63
SLPI	1.07 × 10^−2^	**1.06** × **10^−1^**	−0.71	7.96 × 10^−3^	**8.86** × **10^−2^**	−0.70	1.41 × 10^−2^	**1.26** × **10^−1^**	−0.70	7.91 × 10^−3^	× **10^−2^**	−0.70
SLPI	1.80 × 10^−2^	**1.46** × **10^−1^**	−0.64	1.13 × 10^−2^	**1.10** × **10^−1^**	−0.66	1.74 × 10^−2^	**1.43** × **10^−1^**	−0.64	1.18 × 10^−2^	× **10^−1^**	−0.65
KLF5	1.22 × 10^−2^	**1.15** × **10^−1^**	0.54	1.60 × 10^−2^	**1.36** × **10^−1^**	0.49	1.24 × 10^−2^	**1.16** × **10^−1^**	0.54	1.55 × 10^−2^	× **10^−1^**	0.49
CXCR2	1.26 × 10^−2^	**1.18** × **10^−1^**	0.23	1.30 × 10^−2^	**1.20** × **10^−1^**	0.23	1.25 × 10^−2^	**1.17** × **10^−1^**	0.23	1.34 × 10^−2^	× **10^−1^**	0.23
MGMT	1.28 × 10^−2^	**1.19** × **10^−1^**	−0.30	1.09 × 10^−2^	**1.08** × **10^−1^**	−0.31	1.30 × 10^−2^	**1.20** × **10^−1^**	−0.30	1.09 × 10^−2^	× **10^−1^**	−0.31
MOS	1.32 × 10^−2^	**1.21** × **10^−1^**	0.14	5.84 × 10^−3^	**7.29** × **10^−2^**	0.16	1.24 × 10^−2^	**1.16** × **10^−1^**	0.14	6.22 × 10^−3^	× **10^−2^**	0.16
IL10	1.35 × 10^−2^	**1.23** × **10^−1^**	0.05	1.74 × 10^−2^	**1.43** × **10^−1^**	0.05	1.26 × 10^−2^	**1.17** × **10^−1^**	0.05	1.73 × 10^−2^	× **10^−1^**	0.05
GHRL	1.39 × 10^−2^	**1.26** × **10^−1^**	1.08	1.24 × 10^−2^	**1.17** × **10^−1^**	1.06	1.34 × 10^−2^	**1.22** × **10^−1^**	1.08	1.23 × 10^−2^	× **10^−1^**	1.06
KRT7	1.56 × 10^−2^	**1.35** × **10^−1^**	0.40	1.81 × 10^−2^	**1.47** × **10^−1^**	0.39	1.58 × 10^−2^	**1.35** × **10^−1^**	0.40	1.81 × 10^−2^	× **10^−1^**	0.39
CDKN1A	1.70 × 10^−2^	**1.41** × **10^−1^**	0.25	1.91 × 10^−2^	**1.51** × **10^−1^**	0.24	1.70 × 10^−2^	**1.40** × **10^−1^**	0.24	1.94 × 10^−2^	× **10^−1^**	0.24
CDKN1A	3.48 × 10^−2^	**2.17** × **10^−1^**	0.42	4.19 × 10^−2^	**2.37** × **10^−1^**	0.39	3.34 × 10^−2^	**2.11** × **10^−1^**	0.42	4.17 × 10^−2^	× **10^−1^**	0.39
PDPK1	2.65 × 10^−2^	**1.85** × **10^−1^**	0.17	4.31 × 10^−2^	**2.41** × **10^−1^**	0.15	2.61 × 10^−2^	**1.82** × **10^−1^**	0.17	4.25 × 10^−2^	× **10^−1^**	0.15
PDX1	2.72 × 10^−2^	**1.87** × **10^−1^**	0.06	2.29 × 10^−2^	**1.69** × **10^−1^**	0.06	2.28 × 10^−2^	**1.68** × **10^−1^**	0.06	2.07 × 10^−2^	× **10^−1^**	0.06
HSPB1	3.22 × 10^−2^	**2.07** × **10^−1^**	−0.58	4.43 × 10^−2^	**2.45** × **10^−1^**	−0.55	4.65 × 10^−2^	**2.53** × **10^−1^**	−0.53	4.43 × 10^−2^	× **10^−1^**	−0.55
HSPB1	3.66 × 10^−2^	**2.23** × **10^−1^**	−0.56	3.55 × 10^−2^	**2.17** × **10^−1^**	−0.55	**5.03** × **10^−2^**	**2.65** × **10^−1^**	−0.52	3.63 × 10^−2^	× **10^−1^**	−0.55
HSPB1	3.66 × 10^−2^	**2.23** × **10^−1^**	−0.51	4.63 × 10^−2^	**2.52** × **10^−1^**	−0.48	**5.63** × **10^−2^**	**2.80** × **10^−1^**	−0.46	4.73 × 10^−2^	× **10^−1^**	−0.48
THBSI	3.27 × 10^−2^	**2.08** × **10^−1^**	−0.10	3.86 × 10^−2^	**2.27** × **10^−1^**	−0.10	3.36 × 10^−2^	**2.11** × **10^−1^**	−0.10	3.94 × 10^−2^	× **10^−1^**	−0.10
PTEN	3.30 × 10^−2^	**2.09** × **10^−1^**	0.16	**6.99** × **10^−2^**	**3.12** × **10^−1^**	0.14	3.17 × 10^−2^	**2.04** × **10^−1^**	0.16	**6.90** × **10^−2^**	× **10^−1^**	0.14
LGR5	3.63 × 10^−2^	**2.22** × **10^−1^**	−0.07	3.64 × 10^−2^	**2.20** × **10^−1^**	−0.07	4.22 × 10^−2^	**2.41** × **10^−1^**	−0.07	3.88 × 10^−2^	× **10^−1^**	−0.07
SHH	3.96 × 10^−2^	**2.32** × **10^−1^**	−0.07	2.68 × 10^−2^	**1.85** × **10^−1^**	−0.08	4.82 × 10^−2^	**2.59** × **10^−1^**	−0.07	3.10 × 10^−2^	× **10^−1^**	−0.08
TJP1	3.98 × 10^−2^	**2.33** × **10^−1^**	0.31	4.33 × 10^−2^	**2.41** × **10^−1^**	0.30	4.14 × 10^−2^	**2.39** × **10^−1^**	0.31	4.56 × 10^−2^	× **10^−1^**	0.29
PTGS2	4.02 × 10^−2^	**2.35** × **10^−1^**	0.21	3.90 × 10^−2^	**2.28** × **10^−1^**	0.20	4.00 × 10^−2^	**2.34** × **10^−1^**	0.21	3.73 × 10^−2^	× **10^−1^**	0.21
SOX9	4.48 × 10^−2^	**2.48** × **10^−1^**	−0.29	4.02 × 10^−2^	**2.32** × **10^−1^**	−0.30	4.45 × 10^−2^	**2.48** × **10^−1^**	−0.29	4.04 × 10^−2^	× **10^−1^**	−0.30
CTNNB1	4.53 × 10^−2^	**2.50** × **10^−1^**	0.33	**5.05** × **10^−2^**	**2.63** × **10^−1^**	0.33	4.83 × 10^−2^	**2.59** × **10^−1^**	0.33	**5.31** × **10^−2^**	× **10^−1^**	0.32
MLH1	4.55 × 10^−2^	**2.51** × **10^−1^**	−0.23	**6.82** × **10^−2^**	**3.08** × **10^−1^**	−0.22	4.97 × 10^−2^	**2.63** × **10^−1^**	−0.22	**6.80** × **10^−2^**	× **10^−1^**	−0.22
CDKN1B	4.56 × 10^−2^	**2.51** × **10^−1^**	−0.22	4.90 × 10^−2^	**2.59** × **10^−1^**	−0.22	4.41 × 10^−2^	**2.46** × **10^−1^**	−0.23	4.69 × 10^−2^	× **10^−1^**	−0.22
CXCR4	4.83 × 10^−2^	**2.58** × **10^−1^**	−0.43	**5.72** × **10^−2^**	**2.81** × **10^−1^**	−0.42	**5.00** × **10^−2^**	**2.64** × **10^−1^**	−0.43	**5.77** × **10^−2^**	× **10^−1^**	−0.42
CXCR1	4.98 × 10^−2^	**2.63** × **10^−1^**	0.19	**5.38** × **10^−2^**	**2.72** × **10^−1^**	0.18	4.64 × 10^−2^	**2.53** × **10^−1^**	0.19	**5.18** × **10^−2^**	× **10^−1^**	0.18
KRT14	**5.11** × **10^−2^**	**2.67** × **10^−1^**	0.19	3.65 × 10^−2^	**2.20** × **10^−1^**	0.19	**5.15** × **10^−2^**	**2.68** × **10^−1^**	0.19	3.95 × 10^−2^	× **10^−1^**	0.19

Each column shows the p-value (p), the FDR-corrected p-value (adj. p), and the fold change (FC) obtained in a variant of the database. P-values greater than 5% are shown in bold type.

Greater differences in inference were observed among the genes whose p-value is close to the significance level. These genes have a more subtle differential expression, which can be easily damaged by measurement errors and poor estimation and normalization methods. Thus, the more accurate and careful analysis provided by the proposed methods is especially important for making decisions on the differential expression of these more sensitive genes.

Two replicates of the HSPB1 gene could not be identified as differentially expressed when using both the conventional estimators for the *M_t_* and *A_t_* values and our selection method for the LOWESS *f* parameter. Thus, the estimation of the *M_t_* and *A_t_* values by the proposed estimators was crucial in determining the differential expression of the HSPB1 gene.

The genes PTEN, CTNNB1, MLH1, CXCR4, and CXCR1 could only be identified as differentially expressed when the LOWESS parameter was selected by our proposed method. Particularly, the gene CXCR4 only was determined as differentially expressed when the improved estimators for the *M_t_* and *A_t_* values were also used. In contrast, the gene KRT14 was no longer identified as differentially expressed when the LOWESS *f* parameter was selected by our proposed method.

In the following, we briefly describe the association of those genes with intestinal metaplasia of the stomach according to the literature data:

- HSPB1 (heat-shock protein beta-1, also known as HSP27—heat-shock protein 27): It has a protective role against stress-induced cell damage, and its expression has been considered critical for mucosal protection in the stomach (Ebert et al., 2005). Also, it has been reported as down-regulated in esophageal adenocarcinoma (Lv et al., 2019).
- PTEN (phosphatase and tensin homolog): It has been identified as overexpressed in intestinal metaplasia and is a known marker for tumorigenesis and progression of gastric carcinoma (Yang et al., 2003).
- CTNNB1 (beta-catenin 1): It is a canonical oncogene that has been identified as overexpressed in intestinal metaplasia and gastric adenocarcinomas (Werner et al., 2001; Huang et al., 2018).
- MLH1 (mutL homolog 1): Its expression has been reported as absent or downregulated in intestinal metaplasia, dysplasia, and gastric cancers (Takeda et al., 2012; Hu et al., 2018).
- CXCR4 (chemokine receptor type 4): Its expression has been associated with the staging of gastric cancer, being reduced in the majority of gastrointestinal tumors and significantly higher in patients with advanced stages of gastric cancer (Shibuta et al., 1997; Hannelien et al., 2012; Nikzaban et al., 2014).
- CXCR1 (C-X-C motif chemokine receptor 1): It has been reported to be strongly expressed in gastric carcinoma (Eck et al., 2003; Hannelien et al., 2012).
- KRT14 (keratin 14): It is a squamous cell marker that is down-regulated by CDX2 transfection (Liu et al., 2007). In addition, although it has been determined as significantly overexpressed in intestinal metaplasia by our analysis when the parameter was selected by OLIN, it has been reported as down-regulated in esophageal adenocarcinoma when compared to normal esophagus (Lv et al., 2019).

### Genes Involved in Cancer

By performing a gene enrichment analysis, we identified, at a significance level of 5% (after FDR correction), 31 differentially expressed genes that are involved in cancer. Their respective p-values and fold changes are shown in **Table 3**. We remark that their association with intestinal metaplasia has not been clearly demonstrated yet. Thus, further investigation has to be done to confirm such conclusions.

**Table 3:** Genes belonging to the “pathways in cancer” category identified as differentially expressed between normal and intestinal metaplasia groups at a significance level of 5% (after FDR correction).

Gene	Improved estimation for the *M_t_* and *A_t_* values						Conventional estimation for the *M_t_* and *A_t_* values					
	*f* by our method			*f* by OLIN			*f* by our method			*f* by OLIN		
	p	adj. p	FC	p	adj. p	FC	p	adj. p	FC	p	adj. p	FC
PLD1	4.08 × 10^−7^	6.60 × 10^−5^	1.03	3.54 × 10^−7^	5.86 × 10^−5^	0.99	4.31 × 10^−7^	6.73 × 10^−5^	1.03	3.60 × 10^−7^	5.89 × 10^−5^	0.99
PLD1	2.50 × 10^−6^	2.53 × 10^−4^	0.43	3.49 × 10^−6^	3.32 × 10^−4^	0.42	2.36 × 10^−6^	2.41 × 10^−4^	0.43	3.34 × 10^−6^	3.24 × 10^−4^	0.42
PLD1	9.73 × 10^−5^	4.06 × 10^−3^	0.49	9.90 × 10^−5^	4.14 × 10^−3^	0.49	1.07 × 10^−4^	4.35 × 10^−3^	0.48	1.08 × 10^−4^	4.37 × 10^−3^	0.48
MITF	2.68 × 10^−6^	2.68 × 10^−4^	−0.69	6.38 × 10^−6^	5.19 × 10^−4^	−0.69	2.70 × 10^−6^	2.67 × 10^−4^	−0.68	6.29 × 10^−6^	5.19 × 10^−4^	−0.69
MAX	6.06 × 10^−6^	4.93 × 10^−4^	0.43	7.72 × 10^−6^	6.00 × 10^−4^	0.43	5.26 × 10^−6^	4.37 × 10^−4^	0.43	7.13 × 10^−6^	5.67 × 10^−4^	0.43
MAX	1.61 × 10^−3^	3.10 × 10^−2^	0.35	1.35 × 10^−3^	2.75 × 10^−2^	0.35	1.36 × 10^−3^	2.77 × 10^−2^	0.35	1.31 × 10^−3^	2.68 × 10^−2^	0.35
NOS2	7.08 × 10^−6^	5.52 × 10^−4^	1.37	7.61 × 10^−6^	5.93 × 10^−4^	1.34	6.59 × 10^−6^	5.19 × 10^−4^	1.37	7.28 × 10^−6^	5.76 × 10^−4^	1.34
CDKN2B	8.14 × 10^−6^	6.14 × 10^−4^	0.98	8.41 × 10^−6^	6.38 × 10^−4^	0.97	7.79 × 10^−6^	5.94 × 10^−4^	0.98	8.20 × 10^−6^	6.25 × 10^−4^	0.97
CDKN2B	4.00 × 10^−4^	1.16 × 10^−2^	0.24	5.72 × 10^−4^	1.51 × 10^−2^	0.23	3.33 × 10^−4^	1.01 × 10^−2^	0.24	4.84 × 10^−4^	1.34 × 10^−2^	0.24
VEGFB	1.23 × 10^−5^	8.41 × 10^−4^	−0.95	7.23 × 10^−6^	5.68 × 10^−4^	−0.89	4.36 × 10^−6^	3.78 × 10^−4^	−0.94	6.65 × 10^−6^	5.35 × 10^−4^	−0.89
VEGFB	1.09 × 10^−4^	4.40 × 10^−3^	−0.55	1.05 × 10^−4^	4.32 × 10^−3^	−0.55	1.09 × 10^−4^	4.38 × 10^−3^	−0.54	1.04 × 10^−4^	4.26 × 10^−3^	−0.55
ITGA6	2.80 × 10^−5^	1.60 × 10^−3^	0.63	3.92 × 10^−5^	2.06 × 10^−3^	0.59	2.43 × 10^−5^	1.44 × 10^−3^	0.64	3.63 × 10^−5^	1.96 × 10^−3^	0.59
RXRA	3.03 × 10^−5^	1.71 × 10^−3^	0.25	4.33 × 10^−5^	2.23 × 10^−3^	0.26	3.05 × 10^−5^	1.72 × 10^−3^	0.25	4.76 × 10^−5^	2.39 × 10^−3^	0.25
PIAS3	4.53 × 10^−5^	2.29 × 10^−3^	−0.55	2.93 × 10^−5^	1.68 × 10^−3^	−0.57	4.81 × 10^−5^	2.38 × 10^−3^	−0.55	2.85 × 10^−5^	1.65 × 10^−3^	−0.57
ITGA2	5.24 × 10^−5^	2.53 × 10^−3^	0.48	7.52 × 10^−5^	3.33 × 10^−3^	0.47	5.88 × 10^−5^	2.76 × 10^−3^	0.48	7.43 × 10^−5^	3.30 × 10^−3^	0.47
FZD8	6.00 × 10^−5^	2.83 × 10^−3^	−0.60	5.09 × 10^−5^	2.51 × 10^−3^	−0.60	6.05 × 10^−5^	2.81 × 10^−3^	−0.60	4.83 × 10^−5^	2.42 × 10^−3^	−0.61
FOXO1	1.54 × 10^−4^	5.65 × 10^−3^	−0.53	1.03 × 10^−4^	4.25 × 10^−3^	−0.53	1.39 × 10^−4^	5.24 × 10^−3^	−0.53	1.00 × 10^−4^	4.16 × 10^−3^	−0.54
FOXO1	2.70 × 10^−3^	4.46 × 10^−2^	−0.20	2.66 × 10^−3^	4.33 × 10^−2^	−0.20	2.80 × 10^−3^	4.51 × 10^−2^	−0.20	2.42 × 10^−3^	4.06 × 10^−2^	−0.21
EGLN1	1.85 × 10^−4^	6.42 × 10^−3^	0.50	4.00 × 10^−4^	1.16 × 10^−2^	0.46	1.73 × 10^−4^	6.10 × 10^−3^	0.50	3.96 × 10^−4^	1.16 × 10^−2^	0.46
TGFBR2	2.88 × 10^−4^	9.06 × 10^−3^	−0.36	8.86 × 10^−5^	3.78 × 10^−3^	−0.37	2.68 × 10^−4^	8.46 × 10^−3^	−0.36	8.71 × 10^−5^	3.73 × 10^−3^	−0.37
WNT3	4.16 × 10^−4^	1.19 × 10^−2^	0.51	4.13 × 10^−4^	1.19 × 10^−2^	0.51	4.00 × 10^−4^	1.15 × 10^−2^	0.51	4.22 × 10^−4^	1.21 × 10^−2^	0.50
CKS1B	7.02 × 10^−4^	1.76 × 10^−2^	−0.29	1.91 × 10^−3^	3.46 × 10^−2^	−0.27	1.04 × 10^−3^	2.29 × 10^−2^	−0.27	2.01 × 10^−3^	3.56 × 10^−2^	−0.27
AXIN2	7.63 × 10^−4^	1.88 × 10^−2^	−0.53	8.64 × 10^−4^	2.02 × 10^−2^	−0.53	7.62 × 10^−4^	1.86 × 10^−2^	−0.53	8.52 × 10^−4^	2.01 × 10^−2^	−0.53
CCND1	9.74 × 10^−4^	2.22 × 10^−2^	−0.55	7.00 × 10^−4^	1.75 × 10^−2^	−0.55	9.79 × 10^−4^	2.21 × 10^−2^	−0.55	6.73 × 10^−4^	1.70 × 10^−2^	−0.56
CCND1	3.34 × 10^−3^	**5.12** × **10^−2^**	−0.76	2.81 × 10^−3^	4.51 × 10^−2^	−0.77	3.45 × 10^−3^	**5.19** × **10^−2^**	−0.76	2.88 × 10^−3^	4.58 × 10^−2^	−0.77
CCND1	3.49 × 10^−3^	**5.23** × **10^−2^**	−0.26	4.11 × 10^−3^	**5.80** × **10^−2^**	−0.26	3.19 × 10^−3^	4.95 × 10^−2^	−0.27	3.75 × 10^−3^	**5.45** × **10^−2^**	−0.26
ITGAV	1.03 × 10^−3^	2.30 × 10^−2^	−0.36	1.06 × 10^−3^	2.34 × 10^−2^	−0.35	9.39 × 10^−4^	2.15 × 10^−2^	−0.36	1.04 × 10^−3^	2.29 × 10^−2^	−0.35
CEBPA	1.50 × 10^−3^	2.96 × 10^−2^	0.63	1.79 × 10^−3^	3.32 × 10^−2^	0.60	1.36 × 10^−3^	2.77 × 10^−2^	0.63	1.76 × 10^−3^	3.27 × 10^−2^	0.60
JUN	1.60 × 10^−3^	3.09 × 10^−2^	−0.58	1.57 × 10^−3^	3.04 × 10^−2^	−0.54	1.94 × 10^−3^	3.48 × 10^−2^	−0.56	1.56 × 10^−3^	3.03 × 10^−2^	−0.54
WNT11	2.98 × 10^−3^	4.76 × 10^−2^	0.28	2.96 × 10^−3^	4.65 × 10^−2^	0.28	3.06 × 10^−3^	4.81 × 10^−2^	0.28	2.97 × 10^−3^	4.67 × 10^−2^	0.28
LAMB2	5.18 × 10^−3^	**6.76** × **10^−2^**	−0.52	2.58 × 10^−3^	4.25 × 10^−2^	−0.49	4.42 × 10^−3^	**6.10** × **10^−2^**	−0.49	2.61 × 10^−3^	4.28 × 10^−2^	−0.49

Each column shows the p-value (p), the FDR-corrected p-value (adj. p), and the fold change (FC) obtained in a variant of the database. P-values greater than 5% are shown in bold type.

Particularly, two replicates of the CCND1 gene and the LAMB2 gene were identified as differentially expressed only by the conventional approaches, suggesting that they may be false positives. Next, we briefly describe their association with cancer:

- CCND1 (cyclin D1): In contrast to its underexpression identified by the conventional analyses, it has been frequently reported as overexpressed in intestinal metaplasia, human neoplasias, and several tumors (Hosokawa and Arnold, 1998; Franchi et al., 2015).
- LAMB2 (laminin subunit beta 2): Although its expression has been associated with some carcinomas, ts expression is tightly regulated in normal human tissues and in disease (Wewer et al., 1994; Ljubimova et al., 2006).

## Discussions

Faced with the growing trend of multi-omics data integration in the midst of a replication crisis, improved microarray data analyses are crucial to identifying more reliable results (Ritchie et al., 2015a).

Given that several pixel-level summary statistics are readily available in microarray databases, but are usually discarded in conventional approaches, we propose an improved estimation method for the *M_t_* and *A_t_* values, which takes into account the pixel-level variability. Specifically, we applied the multivariate delta method to derive estimators for the expected values of *M_t_* and *A_t_*, considering their Taylor’s expansion up to the second-order terms. The conventional estimators, nonetheless, approximate the expected values considering only the zeroth-order term. Since the functions that define *M_t_* and *A_t_* are analytic (they are combinations of logarithmic function through addition or subtraction), the higher the number of terms of the Taylor expansion, the better the approximation of the function. Thus, we expect that the proposed estimators provide a better quantification of the hybridization signal. Also, by using these improved estimators, pixel-level dispersion can play an essential role in the analysis, increasing reliability.

To minimize the propagation of errors, the *M_t_* and *A_t_* values have to be properly normalized. Thus, we also propose a method for selecting the LOWESS smoothing parameter *f* that provides an optimal bias–variance compromise, considering some specific characteristics of microarray experiments, such as heteroskedasticity. This optimal normalization method leads to a more parsimonious correction of the systematic biases and, consequently, to greater preservation of the biological variation of interest.

By using the proposed methods, more variability information is considered and retained, improving inferences and preventing false conclusions. Thus, we expect to perform a more conservative analysis, where possibly fewer but more reliable differentially expressed genes are identified. In other words, we expect a reduction in both the false-positive and false-negative error rates.

Besides the theoretical support, relevant empirical observations could be drawn by a comparative study between the methods using real intestinal metaplasia microarray data. The results shows that inferences on differential gene expression were moderately affected by the incorporation of the pixel-level variability in the estimation of the *M_t_* and *A_t_* values and significantly affected by the LOWESS within-slide normalization using a smoothing parameter selected by the method. Both proposed methods tend to increase the within-group variability (the denominator of the t-statistic). However, for many genes, such increase occurred along with an increase in the difference between the group means (the absolute value of the t-statistic numerator), significantly reducing their respective p-values. Thus, many genes were identified as differentially expressed only when the proposed methods were used and some of them have been validated by other studies.

It is important to remark that most of the genes reported in the literature as differentially expressed in intestinal metaplasia were validated with a very strong association with the disease. Thus, these genes are probably more robust to difference approaches for estimating and normalizing the gene expression levels. On the other hand, genes sensitive to methods that address essential uncertainties in measurements are precisely those plagued with major reproducibility issues. Measurement error is one of the most damaging sources of error and has been neglected in many published analyses, thereby increasing uncertainty in parameter estimates and even inflating the estimates of effect sizes (Loken and Gelman, 2017). Thus, particularly for those sensitive genes, a more robust analysis is needed so that false conclusions are not made.

In this paper, we focused on gene expression from two-color microarray data, but it is possible to use the same ideas to improve estimation and normalization of any fluorescent signal quantified by microarray image analysis. Also, the proposed methods could be adapted for oligonucleotide (one-color) microarray data. Particularly, the cyclic LOWESS normalization method (Bolstad et al., 2003) could be extended by just considering that the *M_t_* and *A_t_* values are defined by comparing pairs of arrays instead of pairs of channels and that the LOWESS normalization is applied to all distinct combination of two arrays. Although not so straightforward, it is also possible to adapt our methods to handle next-generation sequencing (NGS) data. Recently, Law et al. (Law et al., 2014) showed that RNA-Seq counts after log transformation and normalization by sequencing depth (log-counts per million, or log-cpm) can be properly analyzed by methods based on the normal distribution if a precision weight for each observation is taken into account. It was used to adapt all methods in the limma package (initially developed for microarrays) to also handle RNA-Seq and other sequence count data (Ritchie et al., 2015b). Therefore, considering the current need for accounting and propagating measurement uncertainties through analyses of NGS data (O’Rawe et al., 2015), a possible future work is to adapt our ideas to improve transcriptome profiling from RNA-Seq data. Specifically, one could investigate whether it is possible to use the delta method for incorporating a measure of uncertainty to each base call, usually provided by base-calling algorithms, into the log-cpm estimator, leading to a more accurate gene expression quantification from RNA-Seq data.

## Data Availability

The omicsMA R package contains the source code of the proposed methods and part of the metaplasia dataset analyzed in this study. It was implemented using R, version 3.5.1, and depends on the locfit (Loader, 2013), maigesPack (Esteves et al., 2016), and limma (Ritchie et al., 2015b) R packages. The omicsMA R package is available at https://github.com/adele/omicsMA, and the latest release is available at https://github.com/adele/omicsMA/releases/latest.

## Ethics Statement

This study was carried out in accordance with the recommendations of the international guidelines for investigations involving human beings with written informed consent from all subjects. All subjects gave written informed consent in accordance with the Declaration of Helsinki. The protocol was approved by the Ethics Institutional Committee of the A.C. Camargo Cancer Center (process number 1023/07).

## Author Contributions

AR and RH conceived of the presented ideas. AR derived the models, implemented the methods, and analyzed the data. AR wrote the manuscript with support from RH and JS. All authors discussed the results and contributed to the final manuscript. RH and JS supervised the project.

## Funding

This study was financed in part by the Coordenação de Aperfeiçoamento de Pessoal de Nível Superior - Brasil (CAPES) - Finance Code 001; National Council of Technological and Scientific Development (CNPq); and NAP eScience – PRP - USP. It was also supported by the Foundation for Research Support of the State of São Paulo (FAPESP) [grants 06/03227-2, 2011/50761-2 and 2015/01587-0].

## Conflict of Interest Statement

The authors declare that the research was conducted in the absence of any commercial or financial relationships that could be construed as a potential conflict of interest.

## Appendix

### Estimation of *E*(*M_tj_*) and *E*(*A_tj_*) by the Delta Method

Let *f* (*R_tj_*, *G_tj_*) be a twice differentiable function of two random variables, *R_tj_* and *G_tj_*.The second-order Taylor’s expansion of at $\left(E(R_{tj}),E(G_{tj})\right)$ is:

$$\begin{array}{l} f(R_{tj},G_{tj})\approx f(E(R_{tj}),E(G_{tj}))+\frac{\partial f}{\partial R_{tj}}(E(R_{tj}),E(G_{tj}))(R_{tj}-E(R_{tj}))+\\ \;\frac{\partial f}{\partial G_{tj}}(E(R_{tj}),E(G_{tj}))(G_{tj}-E(G_{tj}))+\\ \;\frac{1}{2}(\frac{\partial^{2}f}{\partial R_{tj}^{2}}(E(R_{tj}),E(G_{tj})){\left(R_{tj}-E(R_{tj})\right)}^{2}+2\frac{\partial^{2}f}{\partial R_{tj}\partial G_{tj}}(E(R_{tj}),E(G_{tj}))\\ \;[(R_{tj}-E(R_{tj}))(G_{tj}-E(G_{tj}))]+\frac{\partial^{2}f}{\partial G_{tj}^{2}}(E(R_{tj}),E(G_{tj})){\left(G_{tj}-E(G_{tj})\right)}^{2}). \end{array}$$

An approximation of $\left(E(f(R_{tj},G_{tj})\right)$ can be determined by the expected value of the second-order Taylor’s expansion of *f*:

$$\begin{array}{l} E(f(R_{tj},G_{tj}))\approx E[f(E(R_{tj}),E(G_{tj})))]+\frac{\partial f}{\partial R_{tj}}(E(R_{tj}),E(G_{tj}))E(R_{tj}-E(R_{tj}))+\\ \;\frac{\partial f}{\partial G_{tj}}(E(R_{tj}),E(G_{tj}))E(G_{tj}-E(G_{tj}))+\\ \;\frac{1}{2}(\frac{\partial^{2}f}{\partial R_{tj}^{2}}(E(R_{tj}),E(G_{tj}))E[{\left(R_{tj}-E(R_{tj})\right)}^{2}]+\\ \;2\frac{\partial^{2}f}{\partial R_{tj}\partial G_{tj}}(E(R_{tj}),E(G_{tj}))E[(R_{tj}-E(R_{tj}))(G_{tj}-E(G_{tj}))]+\\ \;\frac{\partial^{2}f}{\partial G_{tj}^{2}}(E(R_{tj}),E(G_{tj}))E[{\left(G_{tj}-E(G_{tj})\right)}^{2}])). \end{array}$$

Considering that

$$\begin{array}{l} \text{Var }(R_{tj})=E[{\left(R_{tj}-E(R_{tj})\right)}^{2}],\\ \text{Var }(G_{tj})=E[{\left(G_{tj}-E(G_{tj})\right)}^{2}],\text{ and}\\ Cov\;(R_{tj},G_{tj})=E[(R_{tj}-E(R_{tj}))(G_{tj}-E(G_{tj}))], \end{array}$$

the following simplified expression for the expected value of *f* (*R_tj_*, *G_tj_*) is obtained:

$$\begin{array}{l} E(f(R_{tj},G_{tj}))\approx f(E(R_{tj}),E(G_{tj}))+\frac{1}{2}(\frac{\partial^{2}f}{\partial R_{tj}^{2}}(E(R_{tj}),E(G_{tj}))\text{Var }(R_{tj})+\\ \;2\frac{\partial^{2}f}{\partial R_{tj}\partial G_{tj}}(E(R_{tj}),E(G_{tj}))\text{C}ov\;(R_{tj},G_{tj})+\\ \;\frac{\partial^{2}f}{\partial G_{tj}^{2}}(E(R_{tj}),E(G_{tj}))\text{Var }(G_{tj})). \end{array}$$

Since

$$M_{tj}=f(R_{tj},G_{tj})≐\log_{2}(R_{tj})-\log_{2}(G_{tj}),$$

the first and second derivatives of the function that defines *M_tj_* are:

$$\begin{array}{ccc} \frac{\partial f}{\partial R_{tj}}=\frac{1}{R_{tj}ln\;(2)}; & \frac{\partial f}{\partial G_{tj}}=-\frac{1}{G_{tj}ln\;(2)}; & \\ \frac{\partial^{2}f}{\partial R_{tj}^{2}}=-\frac{1}{R_{tj}^{2}ln\;(2)}; & \frac{\partial^{2}f}{\partial G_{tj}^{2}}=\frac{1}{G_{tj}^{2}ln\;(2)}; & \frac{\partial^{2}f}{\partial R_{tj}\partial G_{tj}}=0. \end{array}$$

Assuming that $E(R_{tj})$ and $E(G_{tj})$ are non-zero, an approximation of $E(M_{tj})=E(\log_{2}(R_{tj})-\log_{2}(G_{tj}))$ can be obtained by using its second-order Taylor’s expansion:

$$\begin{array}{l} E(M_{tj})=E(\log_{2}(R_{tj})-\log_{2}(G_{tj}))\\ \;\approx \log_{2}(E(R_{tj}))-\log_{2}(E(G_{tj}))+\frac{1}{2}\left(-\frac{Var\;(R_{tj})}{ln\;(2)E^{2}(R_{tj})}+\frac{Var\;(G_{tj})}{ln\;(2)E^{2}(G_{tj})}\right)\\ \;=\log_{2}(E(R_{tj}))-\log_{2}(E(G_{tj}))+\frac{1}{2ln\;(2)}\left(-\frac{Var\;(R_{tj})}{E^{2}(R_{tj})}+\frac{Var\;(G_{tj})}{E^{2}(G_{tj})}\right). \end{array}$$

Let the non-zero background-corrected signals be estimators for the expected values of the foreground signals, i.e.,

$$\hat{E}(R_{tj})={\bar{R}}_{tc},\text{ with }{\bar{R}}_{tc}\neq 0,$$

$$\hat{E}(G_{tj})={\bar{G}}_{tc},\text{ with }{\bar{G}}_{tc}\neq 0.$$

Denote the sample variance estimators, obtained across the pixel intensities within each spot, as ${\hat{\sigma }}^{2}(R_{t})$ (for the test channel) and ${\hat{\sigma }}^{2}(G_{t})$ (for the control channel). Also, assume that these estimators do not depend on thebackground correction. We can derive an estimator for $E(M_{tj})$ as follows:

$${\tilde{M}}_{t}≐\log_{2}({\bar{R}}_{tc})-\log_{2}({\bar{G}}_{tc})+\frac{1}{2ln\;(2)}\left(-\frac{{\hat{\sigma }}^{2}(R_{t})}{{\bar{R}}_{tc}^{2}}+\frac{{\hat{\sigma }}^{2}\left(G_{t}\right)}{{\bar{G}}_{tc}^{2}}\right).$$

Since

$$A_{tj}=f(R_{tj},G_{tj})≐\frac{\log_{2}(R_{tj})+\log_{2}(G_{tj})}{2},$$

we can estimate $E(A_{tj})$ in a similar way to $E(M_{tj})$. The first and second derivatives of *A_tj_* are:

$$\begin{array}{l} \frac{\partial f}{\partial R_{tj}}=\frac{1}{2ln\;(2)R_{tj}};\;\frac{\partial f}{\partial G_{tj}}=\frac{1}{2ln\;(2)G_{tj}};\\ \frac{\partial^{2}f}{\partial R_{tj}^{2}}=-\frac{1}{2ln\;(2)R_{tj}^{2}};\;\frac{\partial^{2}f}{\partial G_{tj}^{2}}=-\frac{1}{2ln\;(2)G_{tj}^{2}};\;\frac{\partial^{2}f}{\partial R_{tj}\partial G_{tj}}=0, \end{array}$$

An approximation of $E(A_{tj})$ is obtained by using its second-order Taylor’s expansion:

$$\begin{array}{l} E(A_{tj})\approx \frac{1}{2}(\log_{2}(E(R_{tj}))+\log_{2}(E(G_{tj})))+\frac{1}{2}\left(-\frac{Var\;(R_{tj})}{2ln\;(2)E^{2}(R_{tj})}-\frac{Var\;(G_{tj})}{2ln\;(2)E^{2}\left(G_{tj}\right)}\right)\\ \;=\frac{1}{2}\left(\log_{2}(E(R_{tj}))+\log_{2}(E(G_{tj}))\right)-\frac{1}{4ln\;(2)}\left(\frac{Var\;(R_{tj})}{E^{2}(R_{tj})}+\frac{Var\;(G_{tj})}{E^{2}(G_{tj})}\right). \end{array}$$

Considering the sample estimators of the expected values and variances of *R_tj_* and *G_tj_*, we can derive the following estimator for $E(A_{tj})$:

$${\tilde{A}}_{t}≐\frac{1}{2}\left(\log_{2}({\bar{R}}_{tc})+\log_{2}({\bar{G}}_{tc})\right)-\frac{1}{4ln\;(2)}\left(\frac{{\hat{\sigma }}^{2}(R_{t})}{{\bar{R}}_{tc}^{2}}+\frac{{\hat{\sigma }}^{2}(G_{t})}{{\bar{G}}_{tc}^{2}}\right).$$

### A.2.Estimation of *Var* (*M_tj_*) and *Var* (*A_tj_*) by the Delta Method

We can derive an estimator for Var (*f* (R*_tj_*, G*_tj_*)) by computing the variance of the first-order Taylor’s expansion of *f* (R*_tj_*, G*_tj_*) at $\left(E(R_{tj}),E(G_{tj})\right)$:

$$\begin{array}{l} \text{Var }(f(R_{tj},G_{tj}))\approx {\left(\frac{\partial f}{\partial R_{tj}}(E(R_{tj}),E(G_{tj}))\right)}^{2}\text{Var }(R_{tj})+\\ \;{\left(\frac{\partial f}{\partial G_{tj}}(E(R_{tj}),E(G_{tj}))\right)}^{2}\text{Var }(G_{tj})+\\ \;2\left(\frac{\partial f}{\partial R_{tj}}(E(R_{tj}),E(G_{tj}))\right)\left(\frac{\partial f}{\partial G_{tj}}(E(R_{tj}),E(G_{tj}))\right)Cov\;(R_{tj},G_{tj}). \end{array}$$

The second-order term was not considered because $\text{Var }(R_{tj}^{2})$ and $\text{Var }(G_{tj}^{2})$ cannot be usually estimated.

Since $M_{tj}=f(R_{tj},G_{tj})≐\log_{2}(R_{tj})-\log_{2}(G_{tj})$, with the first and second derivative showed in Appendix 5, we can obtain an approximation of Var (*M_tj_*) as follows:

$$\begin{array}{l} \text{Var }(M_{tj})\approx {\left(\frac{1}{ln\;(2)E(R_{tj})}\right)}^{2}\text{Var }(R_{tj})+{\left(-\frac{1}{ln\;(2)E(G_{tj})}\right)}^{2}\text{Var }(G_{tj})+\\ \;2\left(\frac{1}{ln\;(2)E(R_{tj})}\right)\left(-\frac{1}{ln\;(2)E(G_{tj})}\right)Cov\;(R_{tj},G_{tj})\\ \;=\frac{1}{\ln^{2}(2)}\left(\frac{Var\;(R_{tj})}{E^{2}(R_{tj})}+\frac{Var\;(G_{tj})}{E^{2}(G_{tj})}-2\frac{Cov\;(R_{tj},G_{tj})}{E(R_{tj})E(G_{tj})}\right). \end{array}$$

Consider the sample estimators of the expected values of *R_tj_* and *G_tj_*, denoted by, respectively, ${\bar{R}}_{tc}$ and ${\bar{G}}_{tc}$, and assume that they are non-zero. Also, consider their variance and covariance sample estimators, denoted by, respectively, ${\hat{\sigma }}^{2}(R_{t})$, ${\hat{\sigma }}^{2}(G_{t})$, and $\hat{\sigma }(R_{t},G_{t})$, and assume that they are independent of the background correction. We can derive the following estimator for Var (*M_tj_*) :

$${\hat{\sigma }}^{2}(M_{t})≐\frac{1}{\ln^{2}(2)}\left(\frac{{\hat{\sigma }}^{2}(R_{t})}{{\bar{R}}_{tc}^{2}}+\frac{{\hat{\sigma }}^{2}(G_{t})}{{\bar{G}}_{tc}^{2}}-2\frac{\hat{\sigma }(R_{t},G_{t})}{{\bar{R}}_{tc}{\bar{G}}_{tc}}\right).$$

Considering that *A_tj_* is defined by the function

$$f(R_{tj},G_{tj})≐\frac{\log_{2}(R_{tj})+\log_{2}(G_{tj})}{2},$$

we can estimate Var (*A_tj_*) in a similar way to Var (*M_tj_*).

By using the first and second derivatives of A*_tj_*, which are showed in Appendix (Barrett et al., 2012), we obtain the following approximation of Var (*A_tj_*):

$$\begin{array}{l} \text{Var }(A_{tj})\approx {\left(\frac{1}{2\;ln\;(2)E(R_{tj})}\right)}^{2}\text{Var }(R_{tj})+{\left(-\frac{1}{2\;ln\;(2)E(G_{tj})}\right)}^{2}\text{Var }(G_{tj})+\\ \;2\left(\frac{1}{2\;ln\;(2)E(R_{tj})}\right)\left(-\frac{1}{2\;ln\;(2)E(G_{tj})}\right)\text{C}ov\;(R_{tj},Gt_{tj})\\ \;=\frac{1}{4\;ln^{2}(2)}\left(\frac{Var\;(R_{tj})}{E^{2}(R_{tj})}+\frac{Var\;(G_{tj})}{E^{2}(G_{tj})}+2\frac{\text{C}ov\;(R_{tj},G_{tj})}{E(R_{tj})E(G_{tj})}\right). \end{array}$$

Rewriting the above expression using the sample estimators for the expected value, variance and covariance of *R_tj_* and *G_tj_*, we derive the following estimator for Var (*A_tj_*) :

$${\hat{\sigma }}^{2}(A_{t})≐\frac{1}{4\ln^{2}(2)}\left(\frac{{\hat{\sigma }}^{2}(R_{t})}{{\bar{R}}_{tc}^{2}}+\frac{{\hat{\sigma }}^{2}(G_{t})}{{\bar{G}}_{tc}^{2}}+2\frac{\hat{\sigma }\left(R_{t},G_{t}\right)}{{\bar{R}}_{tc}{\bar{G}}_{tc}}\right).$$
